# Advances in Additive Manufacturing of Composites via Friction Stir Deposition

**DOI:** 10.3390/ma19183922

**Published:** 2026-09-15

**Authors:** Xiaohong Liu, Zhihao Chen, Yunping Li, Zhigao Chen, Hui Wang, Xiaowei Wang, Dongwei Shu

**Affiliations:** 1 State Key Laboratory of Powder Metallurgy, Central South University, Changsha 410083, China; 2 School of Ocean Engineering, Harbin Institute of Technology at Weihai, Weihai 264209, China; 3 School of Aeronautic Engineering, Changsha University of Science and Technology, Changsha 410114, China; 4 College of Mechanical & Energy Engineering, Beijing University of Technology, Beijing 100124, China; 5 School of Mechanical and Aerospace Engineering, Nanyang Technological University, 50 Nanyang Avenue, Singapore 639798, Singapore

**Keywords:** additive friction stir deposition, metal matrix composites, solid-state additive manufacturing, interfacial bonding, microstructural evolution, mechanical properties

## Abstract

The growing demand for large, lightweight, heat-resistant, and multifunctional aerospace structures has raised the requirements for metal matrix composites in terms of defect minimization, performance enhancement, and near-net-shape manufacturing. Additive friction stir deposition of composites enables feedstock delivery, reinforcement mixing, and layer-by-layer consolidation in a thermoplastic state below the melting point of the matrix, thereby mitigating porosity, hot cracking, elemental segregation, reinforcement degradation, and excessive interfacial reactions commonly encountered in fusion-based additive manufacturing. This review summarizes recent advances in the application of this technology to the fabrication of metal matrix composites, elucidates the mechanisms of material flow, interlayer bonding, microstructural evolution, and defect formation during deposition, and discusses the effects of tool design, process parameters, and reinforcement characteristics on interfacial bonding, microstructure control, and mechanical properties. Remaining challenges include the uniform delivery and quantitative control of reinforcements, characterization of interfacial bonding and load transfer, forming stability of complex components, and evaluation of in-service performance; accordingly, thermo-mechanical-flow multiphysics models, multisensor closed-loop control systems, and unified quality-assessment methods should be developed to promote the engineering application of large-scale, multimaterial graded aerospace components.

## 1. Introduction

The development of aerospace equipment has imposed increasingly stringent demands on lightweight structures, load-bearing capability, in-service reliability, and the rapid fabrication of geometrically complex components. Owing to their low density, high specific strength, and tailorable properties, composite materials are promising candidates for load-bearing aerospace structures, thermal-management components, and in-service repair applications. However, fusion-based metal additive manufacturing involves localized melting and rapid solidification during layer-wise deposition, making composite fabrication susceptible to thermal cracking, porosity, elemental segregation, strongly directional solidification microstructures, and residual stresses; furthermore, reinforcement agglomeration and undesirable interfacial reactions can further compromise the processability of composites with high ceramic or nanoscale reinforcement contents. Eliminating the melting–solidification cycle provides an effective route to suppress solidification-induced defects, minimize distortion, and promote the formation of fine equiaxed grains [1,2].

Among typical fusion-based additive manufacturing processes, wire arc additive manufacturing (WAAM) employs an electric arc to melt metallic wire feedstock and deposit material layer by layer, offering advantages such as high deposition efficiency, high material utilization, and suitability for fabricating medium-to-large components, while also enabling localized property tailoring and compositional gradient control through multi-material deposition [3,4]. However, the repeated melting–solidification cycles and relatively high heat input can readily induce residual stresses, distortion, and microstructural heterogeneity, thereby imposing stringent requirements on thermal management and subsequent processing.

Solid-state additive manufacturing avoids complete melting and solidification of the material, thereby suppressing solidification-related defects at their origin and reducing thermal distortion and residual stresses. Among these processes, cold spray additive manufacturing (CSAM) relies on the high-velocity impact of powder particles onto the substrate to induce severe plastic deformation and solid-state interfacial bonding, offering advantages such as limited thermal effects, low oxidation, and high deposition efficiency, and is therefore suitable for coating, repair, and deposition of thermally sensitive materials; however, interparticle porosity, interfacial bonding, and the dimensional accuracy of complex geometries remain constrained by factors such as particle velocity and deposition trajectory [5,6].

In contrast, additive friction stir deposition (AFSD) utilizes frictional heating and severe plastic deformation to induce plasticization, material flow, and interlayer consolidation of continuously supplied feedstock, rather than relying on the high-velocity impact of discrete particles; this mechanism facilitates the fabrication of dense bulk structures with strong interlayer bonding and can promote the fragmentation, migration, and redistribution of reinforcement phases in composites through the stirring action, although further improvements are still required in surface accuracy, complex-path deposition, and tool–material compatibility [7]. Therefore, CSAM is more suitable for low-heat-input coatings and rapid repair, whereas AFSD offers distinct advantages in the fabrication of highly dense structural components, volumetric repair, and the mixing and distribution control of reinforcement phases.

Additive friction stir deposition (AFSD) and friction stir additive manufacturing (FSAM), the latter characterized by layer-by-layer overlapping of sheet materials, utilize frictional heat and severe plastic deformation to achieve solid-state transport, mixing, and metallurgical bonding of metallic materials. The deposition process is essentially a thermomechanically coupled plastic deformation process jointly governed by parameters such as tool rotational speed, feed rate, deposition speed, and layer height, during which the material typically undergoes continuous dynamic recrystallization and develops a fine equiaxed grain structure [8]. Within an appropriate processing window, AFSD can produce nearly fully dense deposits and offers advantages including low heat input, low residual stress, high deposition efficiency, and suitability for the fabrication and repair of large-scale components, thereby showing potential for the rapid manufacturing of aerospace structural components and the remanufacturing of locally damaged regions. However, issues such as sidewall flash, insufficient surface accuracy, and the relatively narrow processing window of high-strength aluminum alloys still limit its capability for fabricating complex components [9].

A variety of material-feeding and deposition configurations have been developed for the solid-state friction additive manufacturing of composites. Friction extrusion additive manufacturing (FEAM) utilizes centrally powder-filled feed rods combined with extrusion deposition for the fabrication of TiC/AA6061 composites [10]; additive friction extrusion deposition (AFED) relies on extrusion-based feeding to fabricate CNT/Al6Mg and nano-Al_2_O_3_/2009Al composites, facilitating reinforcement incorporation, interlayer pore elimination, and the development of near-isotropic properties [11,12]; meanwhile, in-situ friction stir additive repair (IFSAR) is mainly designed for filling and restoring volumetric defects in engineering components [13]. These processes differ in feeding mode, tool configuration, material flow path, and application objective; in this study, they are collectively referred to as solid-state friction additive manufacturing-related technologies, and their composite fabrication mechanisms are compared on the basis of clearly defined process boundaries.

The key to solid-state friction-based additive manufacturing of metal matrix composites is not merely the incorporation of reinforcing phases, but rather ensuring controllable reinforcement content, size, distribution, and interfacial state under high-temperature, large-strain plastic flow conditions. Tool geometry, together with rotational, traverse, and feeding parameters, directly affects the migration, fragmentation, and dispersion of reinforcing phases and governs interlayer bonding, defect formation, and microstructural evolution [14]. Current research is mainly focused on particle-reinforced aluminum alloy systems, while challenges remain, including difficulties in the stable delivery of composite feedstocks, nonuniform reinforcement distribution, unclear interfacial evolution mechanisms, microstructural and property gradients induced by heat accumulation, and insufficient component reproducibility and inter-machine consistency [15]. In addition, evaluations of fatigue, creep, environmental durability, and damage tolerance required for aerospace engineering applications remain limited.

This review summarizes recent advances in solid-state friction additive manufacturing of metal matrix composites, focusing on the underlying processing principles, material systems, reinforcement incorporation strategies, and microstructural evolution mechanisms, while elucidating the effects of processing parameters on defect formation, microstructure, and mechanical properties and highlighting future research directions toward the manufacturing and repair of aerospace components. In contrast to existing reviews that primarily address solid-state additive manufacturing or the general processing principles, process parameters, monitoring, and modeling of AFSD, this review further focuses on composite-specific issues, including reinforcement incorporation, migration and dispersion, interfacial bonding, and microstructure–property relationships, while comparing different solid-state friction-based additive manufacturing routes within a unified process framework. By establishing correlations among feedstock design, material flow, interfacial evolution, defect formation, and property response, and by translating critical evidence gaps into quantifiable directions for future research, this review develops a comprehensive analytical framework for composite process design and engineering-oriented quality assessment.

From a technological perspective, the foundational evidence for AFSD predates the recent research specifically focused on composite materials. Yu et al. systematically elucidated the solid-state deformation characteristics and scalability of AFSD, as well as its distinctions from fusion-based additive manufacturing, from the perspective of non-beam-based metal additive manufacturing [16]; Khodabakhshi and Gerlich further reviewed the technological evolution of friction stir additive manufacturing and its potential for metals and metal matrix composites [17]. Subsequently, Griffiths et al. extended MELD/AFSD to aluminum matrix composites and demonstrated the repair of volumetric defects in 7075 aluminum alloy [18,19], while Phillips et al. established relationships among AFSD process parameters, grain/precipitate evolution, and the mechanical properties of AA6061 [20]. Meanwhile, Rivera et al. systematically demonstrated densification, dynamic recrystallization, and grain refinement induced by high-shear solid-state deposition in Inconel 625 and AA2219 [21,22], while Avery et al. further extended the evaluation to the cyclic fatigue behavior of Inconel 625 and AA7075 [23,24]. Collectively, these early studies provide an important foundation for subsequent investigations into reinforcement-phase transport, interlayer interfaces, microstructural control, and engineering-oriented quality assessment.

This article is a narrative review of advances in solid-state friction-based additive manufacturing of composites and does not involve a meta-analysis; therefore, the full PRISMA systematic review procedure was not followed, although its reporting principles for information sources, search strategies, and study selection were adopted to improve transparency in the literature retrieval and screening. The literature search primarily used the Web of Science Core Collection and Scopus, supplemented by ScienceDirect, SpringerLink, and Google Scholar. The literature search covered publications from 2020 to 2026. Process terms including “additive friction stir deposition (AFSD),” “friction stir additive manufacturing (FSAM),” “friction extrusion additive manufacturing (FEAM),” and “additive friction extrusion deposition (AFED)” were combined with keywords such as “composite,” “metal matrix composite,” and “reinforcement.” Studies directly related to composite fabrication, reinforcement incorporation and dispersion, material flow, interfacial bonding, microstructural evolution, defects, and property regulation were included, with backward reference searching conducted using representative reviews and key papers; duplicates and studies with limited relevance to the technical scope or composite focus were excluded.

## 2. Processing Principles of Additive Friction Stir Deposition for Composites

**Figure 1:**
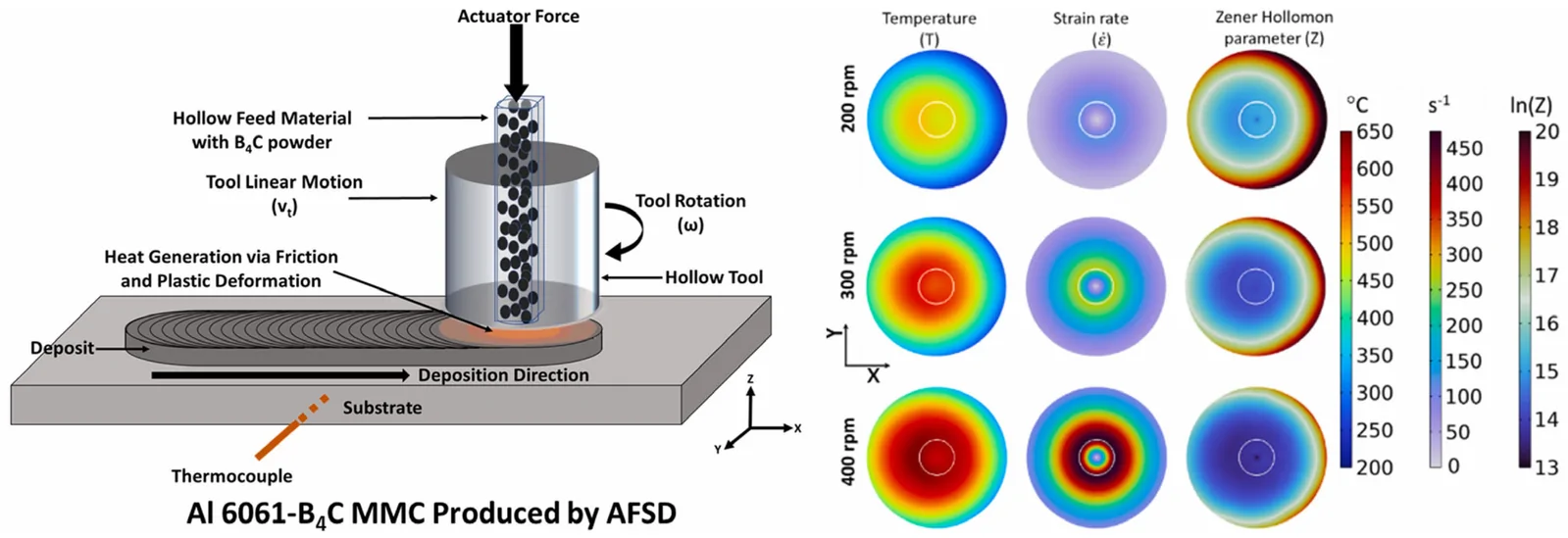
Schematic diagram of the AFSD process and operating-temperature plots under various conditions.

Additive friction stir deposition of composites utilizes frictional heat and heat generated by plastic deformation as the primary heat sources. Under an axial load, feedstocks in the form of rods, powders, wires, or prefabricated composites enter the interaction zone of the rotating tool and undergo heating, shear-induced plasticization, and radial spreading, followed by solid-state bonding with the substrate or the previously deposited layer. The peak deposition temperature is typically 0.5–0.9 times the melting temperature of the material, and its variation is jointly influenced by tool rotational speed, feed rate, axial load, interfacial contact conditions, and material rheological behavior [2], as shown in Figure 1. Therefore, the process is essentially a solid-state deposition process governed by the coupled effects of heat input, plastic flow, and interfacial consolidation.

Fundamental studies further demonstrate that heat input, interfacial contact, plastic flow, and dynamic recrystallization in AFSD are strongly coupled. Garcia et al., through in situ thermal-field monitoring, showed that Cu and Al-Mg-Si exhibit different heat-generation scaling relationships owing to differences in tool–material sliding/sticking conditions [25]; Perry et al. employed X-ray computed tomography (XCT) and microstructural mapping to reveal the non-planar morphology, macroscopic interlocking, and location-dependent recrystallization states at the deposit/substrate interface [26]. Yu and Mishra described AFSD as a metal additive manufacturing route fundamentally governed by severe plastic deformation at elevated temperatures [27], while Griffiths et al. further demonstrated that differences in stacking-fault energy and rheological response among matrix materials can alter the pathways of continuous and discontinuous dynamic recrystallization [28]. Tracer-material-based studies have also directly tracked the material deformation pathway involving extrusion, torsion, and shear thinning, together with the accompanying grain-refinement process [29], while mesh-free thermomechanically coupled models have provided a numerical framework for quantifying temperature, strain rate, and spatial flow gradients [30]. Therefore, the migration and fragmentation of reinforcement phases and their interlayer bonding in composites should be analyzed within the context of the continuous thermo-mechanical-flow history, rather than being interpreted solely on the basis of nominal rotational speed or a single peak temperature.

The different process variants are primarily distinguished by their material-feeding methods. FSAM commonly employs sheet stacking, groove preplacement, or powder spreading and is suitable for localized reinforcement and laminated composite fabrication. AFSD, FEAM, and AFED can employ continuous feeding of rod or powder feedstock, whereas W-FSAM achieves layer-by-layer deposition through continuous wire feeding and can enhance interlayer material exchange and bonding [31]. In FSAM, the tool trajectory and interlayer overlap affect reinforcement entrainment and interfacial bonding [14]. Continuous-feeding processes, however, are prone to flash formation, surface roughness, and heat accumulation [2]. Therefore, the process route should be comprehensively selected according to component geometry, reinforcement morphology, feedstock continuity, and machining allowance.

The feedstock configuration determines the transport stability and initial distribution of the reinforcing phase. Hollow rods can accommodate preplaced particles internally, enabling the simultaneous feeding of the matrix material and reinforcing phase. Krishna et al. employed hollow Al6061 rods filled with B_4_C particles and achieved good interlayer bonding [32]. Ding et al. fabricated Al7075/Al_2_O_3_ composite feedstock rods through powder metallurgy and hot extrusion and achieved dense deposition by adjusting the feed rate [33]. Mixed-powder feeding offers greater flexibility but can readily lead to regional variations in particle distribution and properties [34]. Mixed loading of matrix powder and graphene nanosheets is beneficial for improving the dispersion of nanoscale reinforcements [35].

Prefabricated composite sheets, predrilled rods, powder layers, composite wires, and continuous meshes can also be used to introduce reinforcing phases. Commercial Al-SiC composite feedstock can largely retain its particle size distribution after multilayer deposition, although macroscopic mixing occurs between the bottom deposited layer and the unreinforced substrate [36]. In powder-based additive manufacturing, reversing the rotation direction between adjacent layers can mitigate one-sided material accumulation [37]. Continuous meshes can retain their predefined orientation and structural integrity after deposition [38]. Wire-based W-FSAM has achieved continuous deposition of TiB_2_/7075 and TiC/Al-Cu-Mg composites, while pre-plasticization and thermoplasticization facilitate the fragmentation and redispersion of particle agglomerates [39].

Tool geometry governs the circumferential rotation, radial spreading, and vertical transport of the material and is therefore a key factor in controlling reinforcement dispersion and interlayer bonding. Compared with featureless tools, tools incorporating protrusions, threads, or polygonal features can enhance periodic shearing and downward mixing, thereby improving particle transport and distribution within the deposited layer [40]. However, the effectiveness of the tool is closely related to the rheological characteristics of the matrix alloy, and the same tool geometry does not produce identical particle dispersion in Al-SiC and Cu-SiC systems [40]. In AA7075/ZrO_2_/graphene and AA6061/TiC/grinding-swarf systems, tapered polygonal pins and helical-groove structures can reduce particle agglomeration, refine grains, and improve interlayer bonding [41,42,43]. Therefore, tool design for composite processing should simultaneously consider material transport, reinforcement dispersion, interfacial replasticization, and tool wear.

Tool rotational speed, traverse speed, axial load, and tilt angle jointly determine the heat input per unit volume of material, plastic strain, and dwell time. Increasing the rotational speed generally promotes temperature rise and material flow, whereas excessive heat input may cause grain coarsening, reactions involving the reinforcing phase, or weakening of precipitation strengthening; an excessively high traverse speed, in contrast, can lead to insufficient plasticization and interlayer defects. Previous studies have employed response surface methodology to obtain parameter combinations for different composite systems, such as 1500 r/min, 55 mm/min, and a 3° tilt angle for the AA7075/ZrO_2_/graphene system [44], and 800–1000 r/min, 50 mm/min, and single-pass deposition for the Al6061/SiC system [45,46]. The optimal parameters reported in different studies vary, indicating that the processing window is jointly constrained by the material system, tool geometry, experimental range, and optimization objective and therefore cannot be directly transferred across different systems. Multi-pass overlapping and wide-area deposition can also cause nonuniform thermomechanical effects in the transverse direction. Increasing the overlap width can enhance the compressive action and peak temperature, reduce voids and kissing-bond defects, and improve the interfacial strength between adjacent deposition tracks [47]. In multi-track SiC_p_/Al deposits, the region affected by the feed rod undergoes relatively sufficient shearing and replasticization, whereas interfacial bonding is weaker in the edge regions [48]. Therefore, layer height, overlap width, and deposition path not only affect the dimensional characteristics of the deposit but also determine the extent of material flow across interfaces and replasticization.

The reinforcing phase increases the effective viscosity of the matrix and alters interfacial friction and localized heat generation, resulting in a processing window for composites that is generally narrower than that for monolithic alloys. Excessive reinforcement content can readily cause insufficient material flow, incomplete surface closure, tunnel defects, and tool wear [49]. An appropriate increase in rotational speed can promote material flow and grain-boundary migration across the interface [12], however, excessive heat input makes it difficult to simultaneously maintain particle integrity, interfacial bonding, and microstructural stability of the matrix. Therefore, the key to process control is not simply to increase heat input but to coordinate material plasticization, particle dispersion, cross-interface flow, and microstructural evolution.

Overall, additive friction stir deposition of composites is characterized by low-temperature solid-state processing, flexible feedstock delivery, significant microstructural refinement, and strong interfacial bonding capability, but it also suffers from particle agglomeration, nonuniform distribution, interlayer defects, weak bonding at the edges, and tool wear. Stable deposition relies on the coordinated regulation of feedstock configuration, tool action, material flow, particle dispersion, and interfacial bonding.

## 3. Microstructural Characteristics

**Figure 2:**
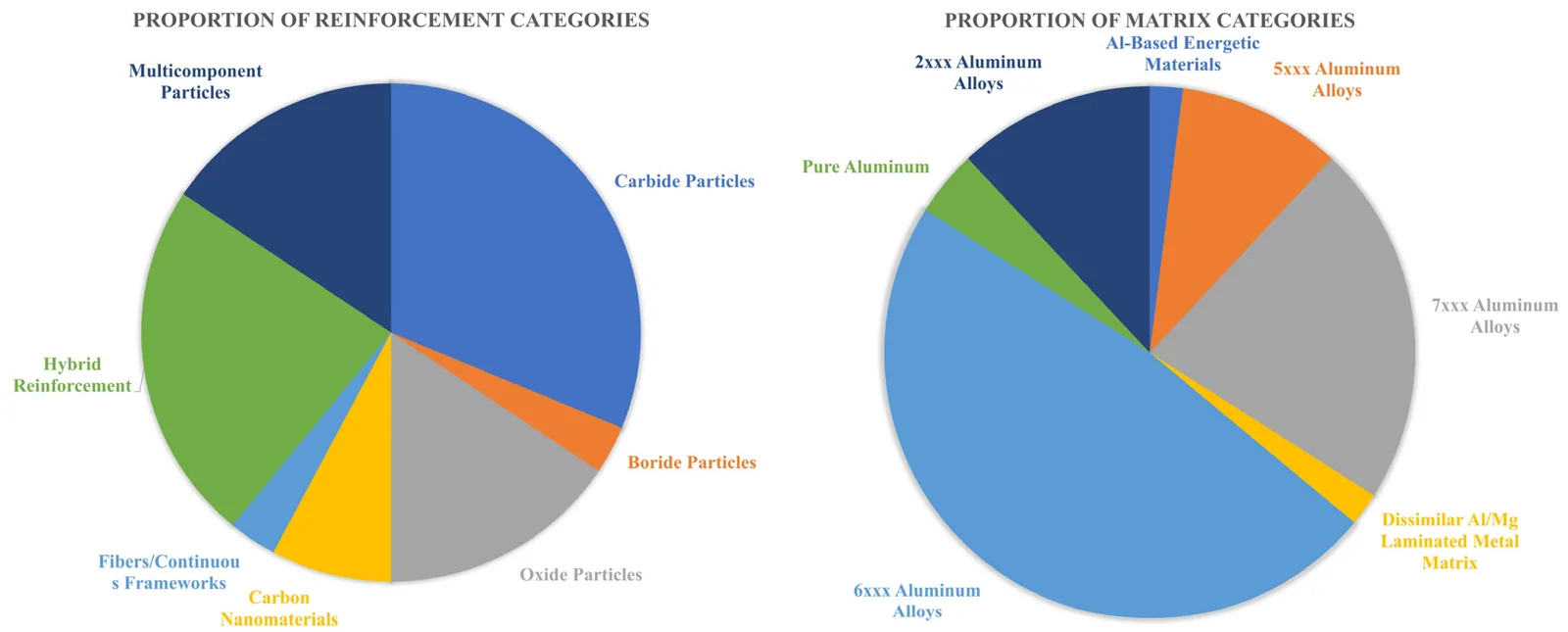
Proportional distribution of matrix materials and reinforcement types reported in existing studies.

Based on reinforcement morphology and functional characteristics, existing composite systems can be broadly categorized into discrete ceramic-particle-reinforced composites, nanocarbon-reinforced composites, fiber- or continuous-mesh-reinforced composites, systems containing reactive or functional fillers, and graded heterogeneous composite architectures. The distribution proportions of matrix materials and reinforcement types reported in existing studies are shown in Figure 2. This classification not only reflects the compositional characteristics of different material systems but is also closely related to the migration, rearrangement, and orientation of the reinforcing phases during additive deposition, as well as the interfacial load-transfer mechanism.

Discrete ceramic particles, represented by SiC, Al_2_O_3_, TiC, and ZrO_2_, constitute the most extensively investigated reinforcement systems. The particles can be introduced through preplaced powders, prefabricated composite rods, or sheet lamination. During deposition, ceramic particles undergo fragmentation and redistribution under intense shear deformation, and an appropriate addition can significantly refine the grains and increase hardness; however, excessive particle content readily induces agglomeration and a sharp loss of ductility, as evidenced by the decrease in elongation from 23.22% to 18.59% when the SiC content increased from 10% to 15% [50]. Compared with the as-cast state, the low-temperature, large-deformation characteristics unique to solid-state deposition can simultaneously improve the strength and ductility of the composite layer and retard the dissolution of precipitates in heat-treatable matrices [51]. For wear-resistant layers with high reinforcement contents, particle fragmentation, overlap interfaces, and transverse heterogeneity become more pronounced; optimizing the overlap width to 5 mm can eliminate voids and kissing-bond defects [47], while multi-pass deposition significantly affects hardness and wear behavior [45,46]. Although sheet lamination can achieve localized reinforcement and equiaxed grain refinement, nonuniform particle distribution and residual microporosity indicate that macroscopic bonding does not guarantee microscopically uniform dispersion [52]. 7xxx-series high-strength aluminum alloys are more sensitive to thermal cycling and tool wear, and SiC contents above 5 vol.% may induce restricted material flow and pin fracture [49], whereas the use of TiC or pre-composited feedstocks can reduce tool mass loss while maintaining mechanical properties and corrosion resistance [53,54].

Nanocarbon materials, including CNTs, graphene, and GNPs, provide dislocation pinning, load transfer, and self-lubricating functions, but their high specific surface area greatly increases the difficulty of controlling dispersion and interfacial reactions.

When introduced through eccentric-hole filling or predeposited layers, these reinforcements can produce submicrometer equiaxed grains and strengths exceeding 450 MPa, together with improved corrosion resistance [55,56]. However, reinforcement efficiency strongly depends on dispersion quality, and a GNP content above 0.8 wt.% is required to produce a distinct hardness increase [35], while agglomeration remains a key bottleneck limiting ductility enhancement. Fibers and continuous meshes extend load-transfer and crack-deflection pathways by retaining a high aspect ratio or predefined orientation. After being sheared into micro- and nanoscale fragments during deposition, basalt fibers bond with the matrix through an interfacial layer on the order of 10 nm, enabling a strength–ductility synergy [57]. Preplaced steel meshes at 12.3 vol.% can simultaneously improve tensile and flexural strength without sacrificing ductility [38], although such systems are highly sensitive to orientation retention and interfacial continuity. Reactive/functional fillers and hybrid reinforcement systems are designed to achieve complementary functions, but their composition ranges and processing windows are narrower. Industrial by-products or energetic fillers can reduce raw-material costs or introduce energy-release characteristics, but compositional fluctuations and interfacial reactions require additional control [58,59]. Hybrid reinforcement combines hard phases, lubricating phases, or corrosion-inhibiting phases to simultaneously optimize mechanical, tribological, and corrosion properties; for example, MoS_2_–diamond and ZrO_2_/Gr systems exhibit favorable overall responses under specific processing parameters [60,61], whereas deviations from these conditions can lead to agglomeration and brittle fracture. Representative composite systems and their principal processing challenges are summarized in Table 1.

Different reinforcement incorporation methods and their applicable ranges are summarized in Table 2.

From the perspective of functional attributes and intended applications, existing composite systems can be further classified into four categories. Wear-resistant/tribological composites primarily include systems reinforced with hard particles such as SiC, ZrO_2_, Al_2_O_3_, and TiO_2_/CeO_2_, with the design objectives of increasing hardness, reducing wear, and improving surface service performance; however, excessive particle contents can readily lead to agglomeration, reduced ductility, and increased tool wear [50,62,63]. Corrosion-resistant systems mainly include CNT/2A12, Al_2_O_3_/7075, and ZrB_2_/6061 composites, in which synergistic improvements in mechanical properties and corrosion resistance are achieved through grain refinement, precipitation control, or enhanced passivation behavior; however, these effects are highly sensitive to the reinforcement content and dispersion state [54,56]. Mechanically tailored composites emphasize the coordinated regulation of strength, ductility, hardness, and anisotropy, with GNPs, TiC, basalt fibers, and continuous steel meshes improving the overall mechanical performance through particle strengthening, load transfer, crack deflection, and directional load bearing, respectively [38,57]. Multifunctional/special-function composites further incorporate capabilities such as radiation shielding, energy release, or in-situ manufacturing for space applications; for example, Al/MgB_2_ combines wear resistance with proton-shielding capability, Al/Ni/CuO integrates structural strength with exothermic functionality, and lunar-regolith-simulant-reinforced AA6061 demonstrates potential for in-situ space resource utilization [58,66,67].

**Figure 3:**
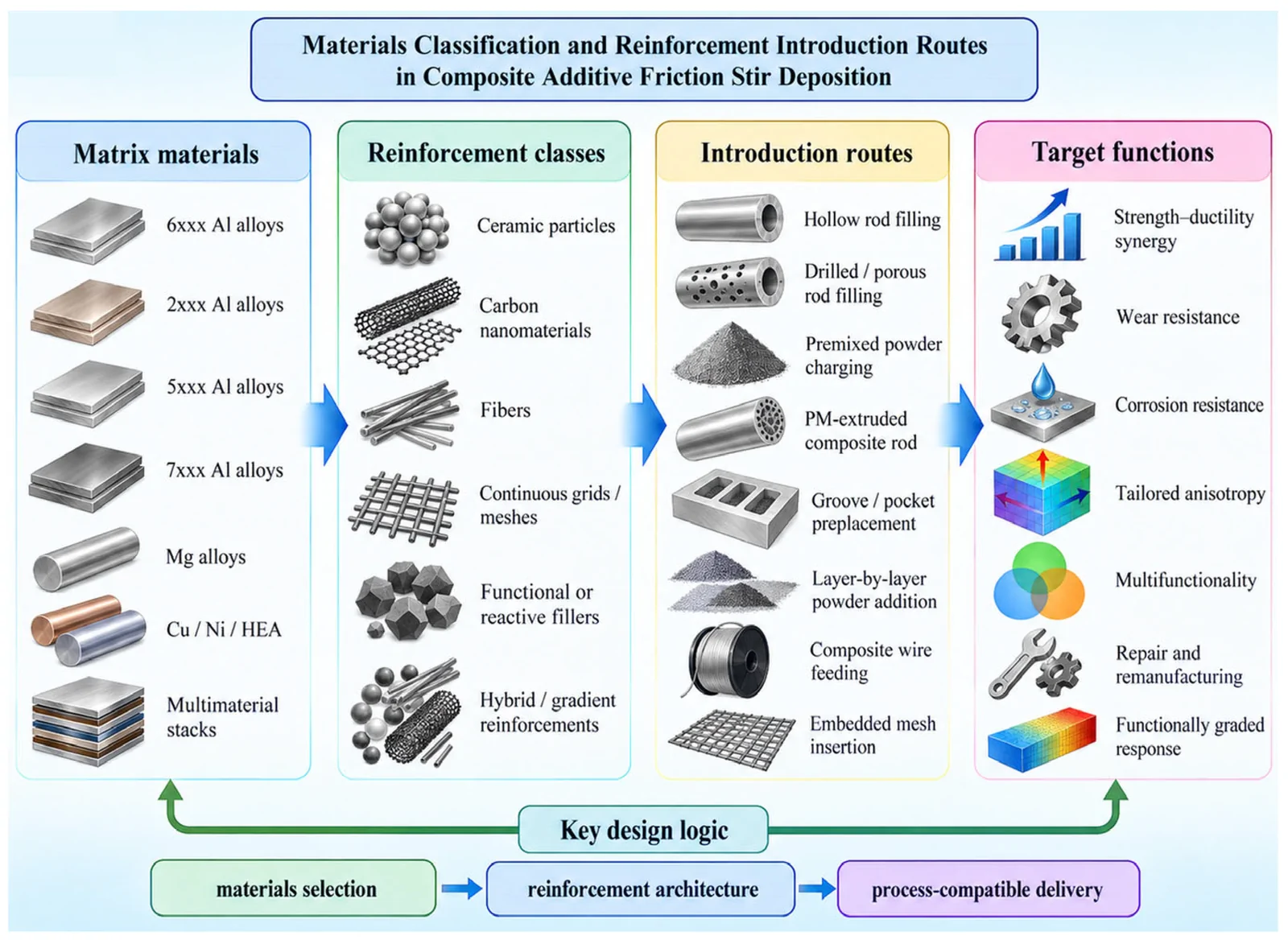
Material–process–function classification of composite friction stir deposition.

Graded heterogeneous composite structures demonstrate the potential of the process for spatial compositional control and functional tailoring. The design of composite materials depends not only on the type of reinforcement but also on multiple factors, including feedstock form, feeding compatibility, and the intended service functions. The relationships among material systems, reinforcement morphologies, feeding methods, and target functions are illustrated in Figure 3. By precisely controlling the reinforcement content and local thermal history, continuously varying mechanical responses can be established within a component, while differences in particle size can also be exploited to induce graded microstructures [69,70]. Such systems require the coordinated integration of compositional control and thermomechanical processing, thereby placing higher demands on equipment control accuracy as well as process stability and robustness.

In summary, particle-reinforced systems have relatively mature processing windows, whereas further advances are required in the dispersion and interfacial characterization of nanocarbon materials; fiber/mesh-reinforced systems are highly sensitive to reinforcement orientation and interfacial continuity, while graded and functional-filler systems require attention to coupled composition–thermal-history design. Different systems cannot be ranked using a single criterion and must instead be comprehensively evaluated in terms of reinforcement integrity, interlayer bonding, ductility retention, and functional requirements; moreover, current evidence remains concentrated on particle-reinforced aluminum-matrix systems, while consistency evaluations across different materials and equipment remain insufficient [15].

## 4. Material-Flow, Interfacial-Bonding, Microstructural-Evolution, and Defect-Formation Mechanisms During Deposition

Material flow during composite deposition exhibits three-dimensional nonuniformity and spatial dependence, and the cross-sections of multilayer deposits also show pronounced interlayer and spatial variations in microstructure.

Lopez et al. used SiO_2_ particles as tracer phases and found that larger angular particles were retained in the center of the deposit, whereas particles near the edges and top experienced more intense rotation, shear, and impact from tool protrusions [34]. Garcia et al. obtained different particle-dispersion characteristics in Al and Cu matrices, indicating that the effect of tool geometry is constrained by the rheological behavior of the matrix [40]. A tapered octagonal pin can improve plastic flow and interlayer bonding in AA7075/ZrO_2_/Gr composites [43]. In W-FSAM, the pre-plasticization and thermoplasticization stages can refine and redisperse TiC agglomerates [39]. Therefore, particle migration should be characterized in conjunction with the local velocity field, residence time, and particle–tool contact frequency.

Fragmentation of the reinforcing phase is beneficial for improving dispersion uniformity, but changes in its size, morphology, and orientation also affect the strengthening mechanisms and anisotropy. After Al-SiC deposition, the characteristic particle size decreases significantly, and nonspherical particles exhibit position-dependent orientations [36]. In SiCp wear-resistant layers, both the major and minor particle axes decrease, while good wear resistance is still maintained [71]. Basalt fibers can be sheared into micrometer- and nanometer-scale fragments, with the former promoting particle-stimulated nucleation and the latter enhancing dislocation storage [57]. TiC can be refined to the nanoscale and form semicoherent interfaces [39]. Therefore, finer reinforcing phases are not necessarily always preferable, and their fragmentation scale, interparticle spacing, and interfacial structure should be matched with mechanisms such as particle-stimulated nucleation, Zener pinning, and Orowan strengthening. Dynamic recrystallization is the dominant feature of microstructural evolution during deposition, and its extent depends on reinforcement particle size, content, and distribution. Grain coarsening after recrystallization may occur around large Al_2_O_3_ particles, whereas fine particles favor the formation of fine equiaxed grains [70]. An appropriate amount of SiC can increase nucleation sites and pin grain boundaries, whereas agglomeration reduces ductility [50]. AA5083/AA6061/SiC, particle-stimulated nucleation and Zener–Hollomon conditions synergistically promote recrystallization and weaken the crystallographic texture [72]. For heat-treatable aluminum alloys, the thermomechanical cycles during deposition can also induce precipitate dissolution, coarsening, and reprecipitation. The β″ phase in Al6061 dissolves during deposition and causes a reduction in strength, while subsequent solution treatment and aging can restore the properties [32,51]. Reinforcing particles can suppress precipitate coarsening and thermal-cycle-induced softening [54]. Therefore, grain refinement, precipitation strengthening, and particle strengthening should be quantitatively evaluated separately.

Interfacial bonding includes particle/matrix, deposit/substrate, and interlayer interfaces. Short-duration solid-state thermal cycles can suppress detrimental reaction phases, enabling the interfacial shear strength of AA2014/SiC to approach that of the matrix [68]. An approximately 7 nm elemental gradient layer at the basalt fiber/AA6061 interface indicates that diffusion contributes to interfacial bonding [57]. A transient liquid phase formed by Zn can improve Al_2_O_3_ wettability and deposit densification, although its duration and volume fraction still require quantitative verification [33]. Interlayer bonding mainly depends on the replasticization depth of the previously deposited layer and material exchange across the interface. Increasing the rotational speed or overlap width, or introducing vertical stirring, can enhance interfacial dynamic recrystallization, grain-boundary migration, and mechanical mixing, thereby suppressing layered porosity, kissing-bond defects, and build-direction anisotropy [12,47,48]. In W-FSAM, the vertical material exchange and interlayer mixing induced by the eccentric tool can further improve interlayer bonding and help reduce anisotropy along the build direction [31]. However, particle orientation, repeated thermal cycling, and microstructural gradients along the build height can still cause property variations [36,66,72,73].

Defect formation generally results from the coupled effects of insufficient plastic flow, particle agglomeration, and increased tool loading. A high reinforcement content impedes matrix flow and can induce incomplete closure, tunnel defects, insufficient interfacial filling, and even tool failure [49]. An insufficient peak temperature or inadequate plasticization can readily lead to voids, weak bonding, and localized agglomeration [10]. Therefore, the absence of macroscopic porosity does not necessarily imply uniformity at the particle scale, and defect evaluation should simultaneously cover volumetric porosity, interfacial continuity, particle spatial distribution, and local mechanical response [34]. Both reinforcement content and processing parameters have critical ranges: moderate increases can improve dispersion and enhance strength, hardness, or corrosion resistance, whereas exceeding the threshold leads to property degradation due to agglomeration and restricted material flow [50,61,74,75]. The formation conditions, effects, and mitigation strategies of common defects are summarized in Table 3.

From the perspective of industrial application, the selection of highly wear-resistant tools should consider both initial cost and service life. WC-Co cemented carbide offers relatively low manufacturing cost, good toughness, and flexibility in tool-geometry machining, making it suitable for composites with low-to-moderate reinforcement contents and for small-batch production; however, pronounced wear may still occur in highly abrasive systems containing high fractions of SiCp. PCD tools exhibit higher hardness and wear resistance, which can reduce tool-geometry degradation and the frequency of tool replacement; however, they involve higher manufacturing costs, and the fabrication and maintenance of complex tool geometries remain relatively limited. Therefore, under industrial conditions, tool selection should not be based solely on the unit price or absolute service life of an individual tool, but should instead be evaluated in terms of life-cycle cost per unit deposition length or per qualified component, while accounting for tool procurement and repair costs, downtime associated with tool replacement, dimensional stability, contamination from tool materials, and the risk of component rejection. For systems with high reinforcement contents, long-duration continuous production, and high-value components, the higher initial investment in tooling may be offset by extended service life and improved process stability; by contrast, under small-batch or low-wear conditions, lower-cost tools such as WC-Co may still offer better overall economic efficiency. At present, systematic quantitative data on tool life and cost in AFSD of composites remain limited, and future studies should establish quantitative relationships among tool-wear rate, service life, and unit-deposition cost.

Overall, existing studies have relatively well established the correlations among grain size, precipitates, fracture morphology, and macroscopic properties, but direct causal evidence for three-dimensional particle migration, transient fragmentation, interlayer mixing, and interface formation remains insufficient. Future studies should combine interrupted experiments using tracer particles, synchronized temperature–load–torque monitoring, three-dimensional particle statistics, and EBSD/TEM characterization, while incorporating the temperature field, flow field, recrystallization, and precipitate evolution into a unified model to distinguish thermal, strain, and tool-contact effects and thereby improve the verifiability and predictive capability of mechanistic interpretations.

## 5. Effects of Processing Parameters on Forming Quality, Microstructure, and Mechanical Properties and Their Regulation Methods

The essence of process-parameter control in solid-state additive manufacturing is to coordinate heat input, strain rate, material flow, and interfacial pressure rather than independently pursue an extreme value of any single parameter. Increasing the rotational speed generally promotes material plasticization and reinforcement mixing, whereas excessive heat input may cause grain coarsening, precipitate dissolution, and reinforcement segregation; feed rate and traverse speed jointly affect the feedstock supplied per unit volume, material residence time, and interlayer heat accumulation. Therefore, the processing window should be comprehensively determined by considering matrix rheological characteristics, reinforcement type, tool geometry, deposition scale, and interlayer thermal history [9]. From an engineering perspective, the heat input per unit deposition length, (*q* = 2*πMω*/(60*v*)), can be used to characterize the combined thermal effects of spindle torque (*M*), tool rotational speed (*ω*), and travel speed (*v*), thereby enabling comparison of the relative heat-input levels among different parameter combinations. On this basis, tool rotational speed and travel speed can be further correlated with temperature response, material flow, and defect characteristics to construct a process map, providing an intuitive basis for identifying and optimizing a stable processing window. For example, the AA5083/MoS_2_-diamond system exhibited relatively favorable overall properties at approximately 800 r/min and 5 mm/min [60]. In the AA6061/TiC-graphene system, increasing the rotational speed from 1200 to 1500 r/min resulted in slight grain growth and pronounced dissolution of Mg-rich precipitates, accompanied by decreases in hardness and wear resistance but a lower corrosion rate [76]. The B_4_C/graphene system exhibited relatively uniform dispersion at 1400 r/min, whereas agglomeration became more severe at 1600 r/min [74]. These results indicate that increasing heat input does not affect strength, ductility, wear resistance, and corrosion resistance in the same manner and therefore cannot be summarized by a single “optimal temperature.” The effects and applicable ranges of the main parameters on heat input, material flow, and forming quality are summarized in Table 4.

Tool geometry determines material-exchange pathways and local shear patterns and can therefore produce markedly different microstructures and properties under the same nominal processing parameters. In the AA7075-O/ZrO_2_/graphene system, a tapered triangular pin exhibited relatively favorable hardness, tensile, compressive, wear, and corrosion properties [41]. In the AA6061/TiC/graphene system, a tapered octagonal pin with helical grooves was more favorable for reinforcement dispersion and improvement of mechanical properties [42]. Another study on AA7075/ZrO_2_/graphene showed that a tapered octagonal pin could reduce wear and improve interlayer bonding [43]. These differences indicate that coupling relationships exist among the number of pin facets, helical grooves, shoulder structure, and material rheological behavior, and these factors should therefore be systematically matched. Meanwhile, axial load, traverse speed, tilt angle, shoulder-to-pin ratio, and overlap width mainly regulate trailing-side filling, cross-layer replasticization, and interfacial consolidation. Analysis of variance showed that rotational speed and shoulder-to-pin ratio made relatively large contributions to the strength and hardness of Al7075/graphene/B_4_C composites, whereas axial force and tilt angle were more critical for stable material flow and defect suppression [77]. Increasing the overlap width can increase the interfacial tensile strength from 155 MPa to 297 MPa [47], although nonuniform effective pressure between the center and edge regions and corresponding property gradients may still exist during multi-track deposition [48].

**Figure 4:**
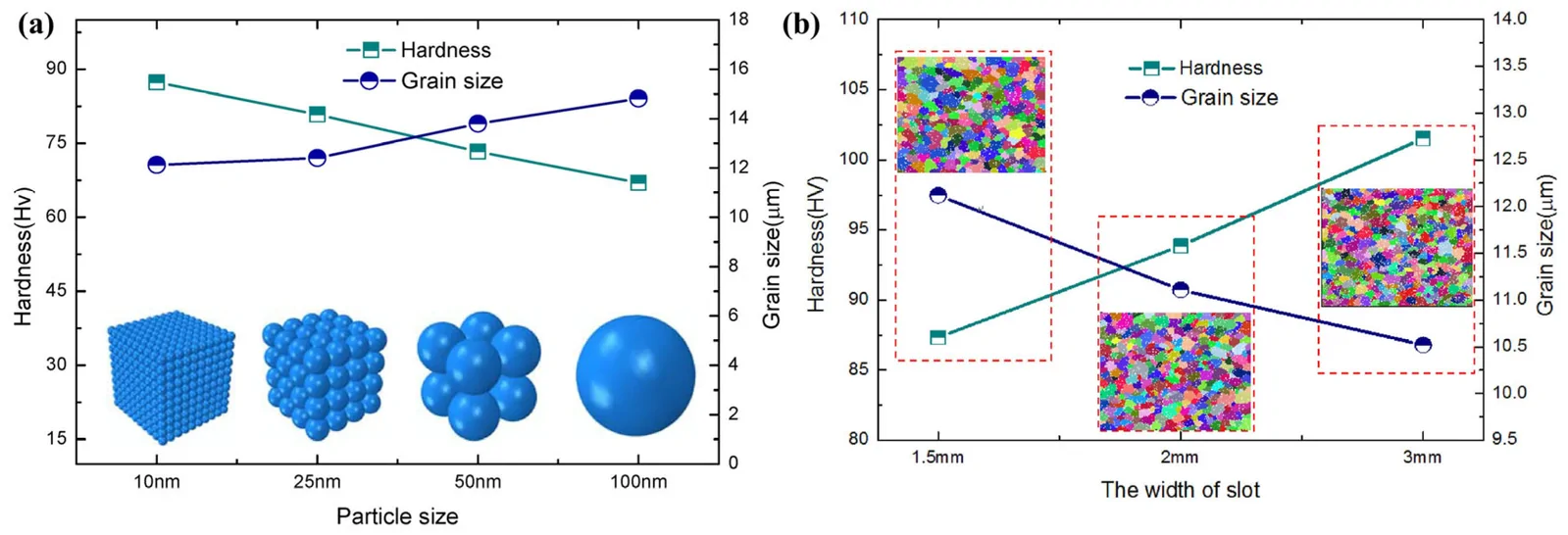
Effects of nanoparticle size and volume fraction on average grain size and hardness, (**a**) nanoparticle size, (**b**) volume fraction.

Reinforcement content and size are important material constraints in process-parameter optimization. The ZrB_2_/6061 system exhibited the best overall properties at 6 vol.%, whereas increasing the reinforcement content to 8 vol.% resulted in decreases in hardness and corrosion resistance due to agglomeration [75]. In SiC/Al6061, a content of 10% is favorable for retaining elongation, whereas 15% increases hardness and strength but significantly reduces ductility [50]. Reducing the Al_2_O_3_ particle size from 100 nm to 10 nm can refine the grains and increase hardness, and increasing the volume fraction produces a similar effect [78], as shown in Figure 4. A distinct strengthening effect is observed only when the GNP content exceeds 0.8 wt.% [35]. In addition, 40 nm TiC can achieve simultaneous strengthening and toughening, whereas 1–4 μm TiC readily induces voids and interfacial debonding because of thermal-expansion mismatch [13]. Therefore, reinforcement design should simultaneously consider dispersion stability, interfacial bonding, load transfer, and the risk of tool wear, rather than determining the addition amount solely according to the intrinsic hardness of the reinforcing phase.

Subsequent heat treatment can restore precipitation strengthening and reduce microstructural gradients, although its effectiveness is constrained by the alloy condition and component scale. After heat treatment, TiC/AA6061 exhibits hardness and strength exceeding those of the matrix, while some of the microstructural gradients along the build height are reduced [10]. After solution treatment and aging, the strength of Al6061-B_4_C recovers to approximately 290 MPa, although specimens deposited at low rotational speed exhibit abnormal grain growth due to secondary-particle coarsening [32]. After solution treatment and aging, the hardness of Al-SiC deposits recovers to the feedstock level without the formation of cracks or pores [36]. Subsequent heat treatment of TiC/Al7075 can simultaneously increase hardness, strength, and elongation, but it cannot eliminate tool wear and particle agglomeration in SiC-containing systems [53]. For large repaired components or pre-aged substrates, bulk furnace heat treatment is also limited by component size, deformation control, and engineering feasibility.

**Figure 5:**
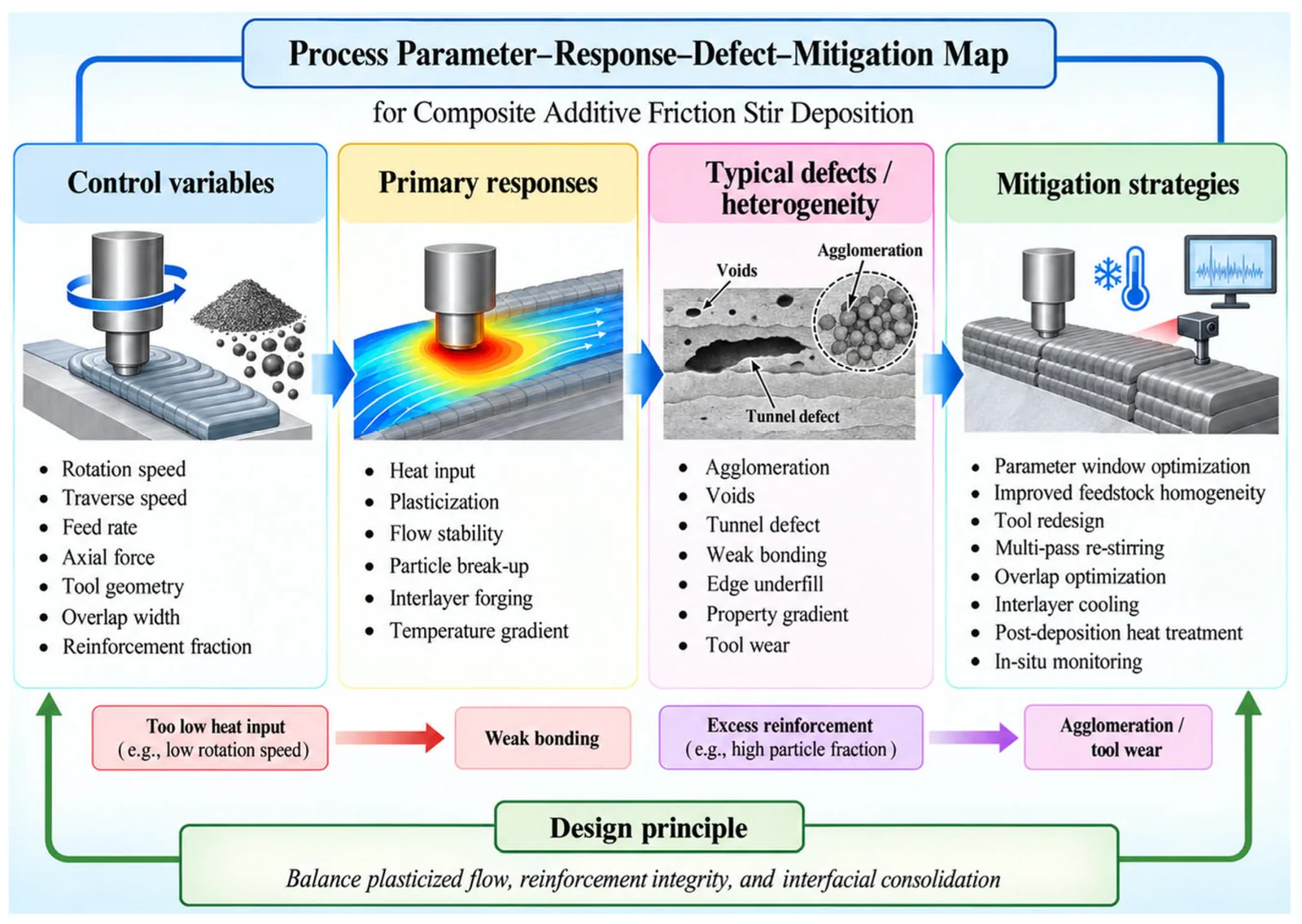
Parameter–defect–mitigation relationships in composite friction stir deposition.

Considering the combined effects of rotational speed, traverse/feed speed, tool geometry, reinforcement content, and post-deposition heat treatment, variation in a single parameter often produces coupled effects on heat input, degree of plasticization, material-flow stability, and interfacial consolidation. To intuitively reveal defect-formation pathways and corresponding regulation strategies when process parameters deviate from the appropriate window, this review establishes a “control variable–process response–typical defect/heterogeneity–mitigation strategy” relationship, as shown in Figure 5.

**Figure 6:**
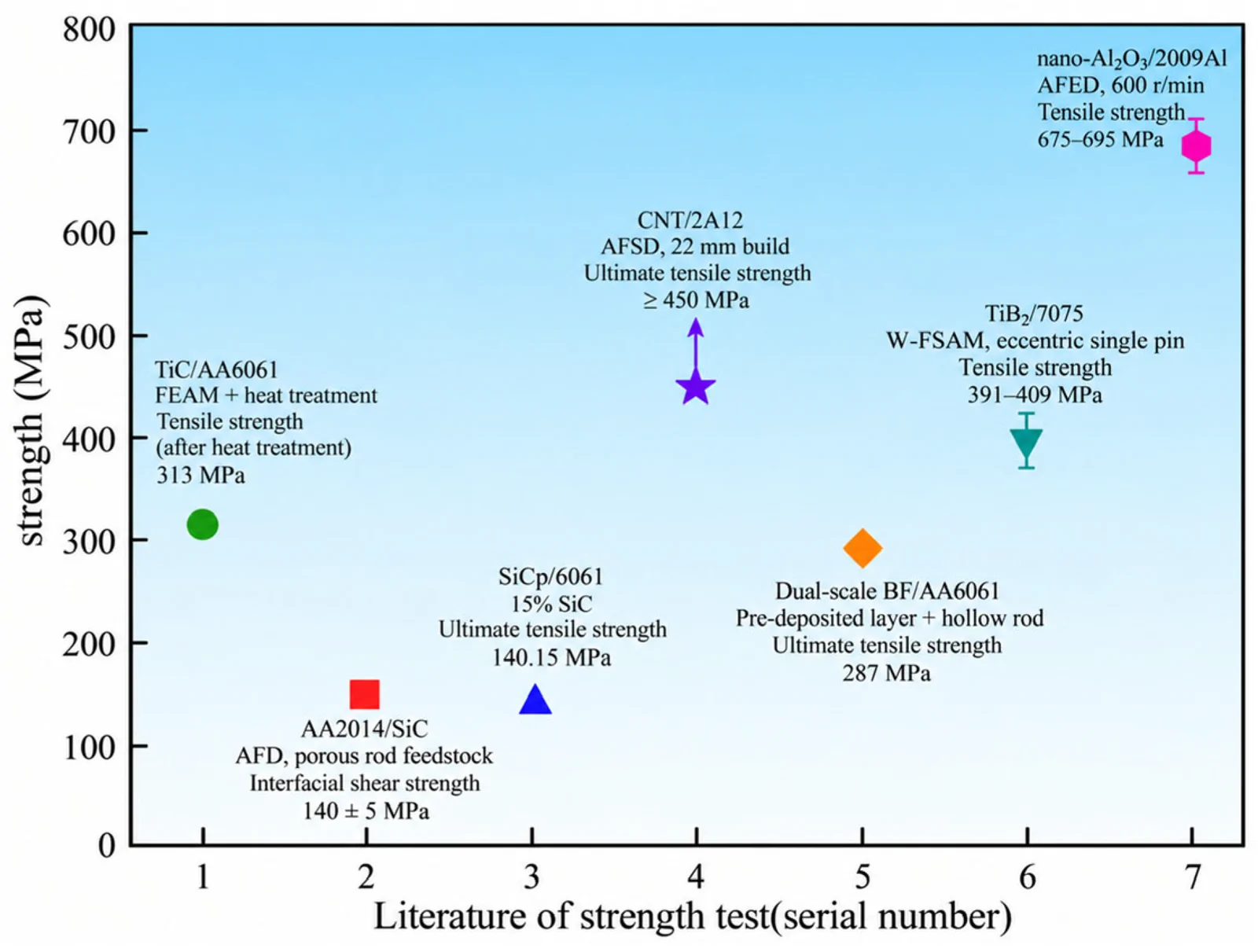
Performance results of representative composite systems.

Trade-offs commonly exist among strength and ductility, wear resistance and corrosion resistance, and hardness and interfacial integrity as a function of process and material parameters. The hardness of 6061-ZrO_2_ composites is approximately twice that of unreinforced AFSD 6061, and the tensile strength is approximately 1.4 times higher, although elongation decreases [62]. By optimizing particle dispersion and precipitate stability, Al_2_O_3_/7075 can improve corrosion resistance while maintaining relatively high strength [54]. CNT/2A12 achieves a combination of relatively high strength and ductility together with a low corrosion current density [56]. Dual-scale basalt-fiber/AA6061 achieves a balance between strength and an elongation of approximately 27% through hierarchical load bearing and crack suppression by micro- and nanoscale reinforcements [57]. Tribological and corrosion properties also exhibit different sensitivities to microstructural variables: TiO_2_ mainly improves hardness and wear resistance, whereas CeO_2_ primarily enhances passivation behavior, although excessive contents lead to agglomeration [64]. In the CeO_2_/B_4_C system, B_4_C bears the applied load, while weakly bonded Ce-containing phases and their lubricating effect contribute to wear reduction, and CeO_2_ can also enhance cathodic inhibition [79]. In CNT/AA6061, the corrosion potential shifts in the positive direction while the corrosion current density increases, indicating that corrosion-resistance evaluation must clearly define the assessment metric and corrosion stage [55]. The quantitative performance results of representative systems are shown in Figure 6, while the corresponding performance trade-offs are summarized in Table 5.

**Figure 7:**
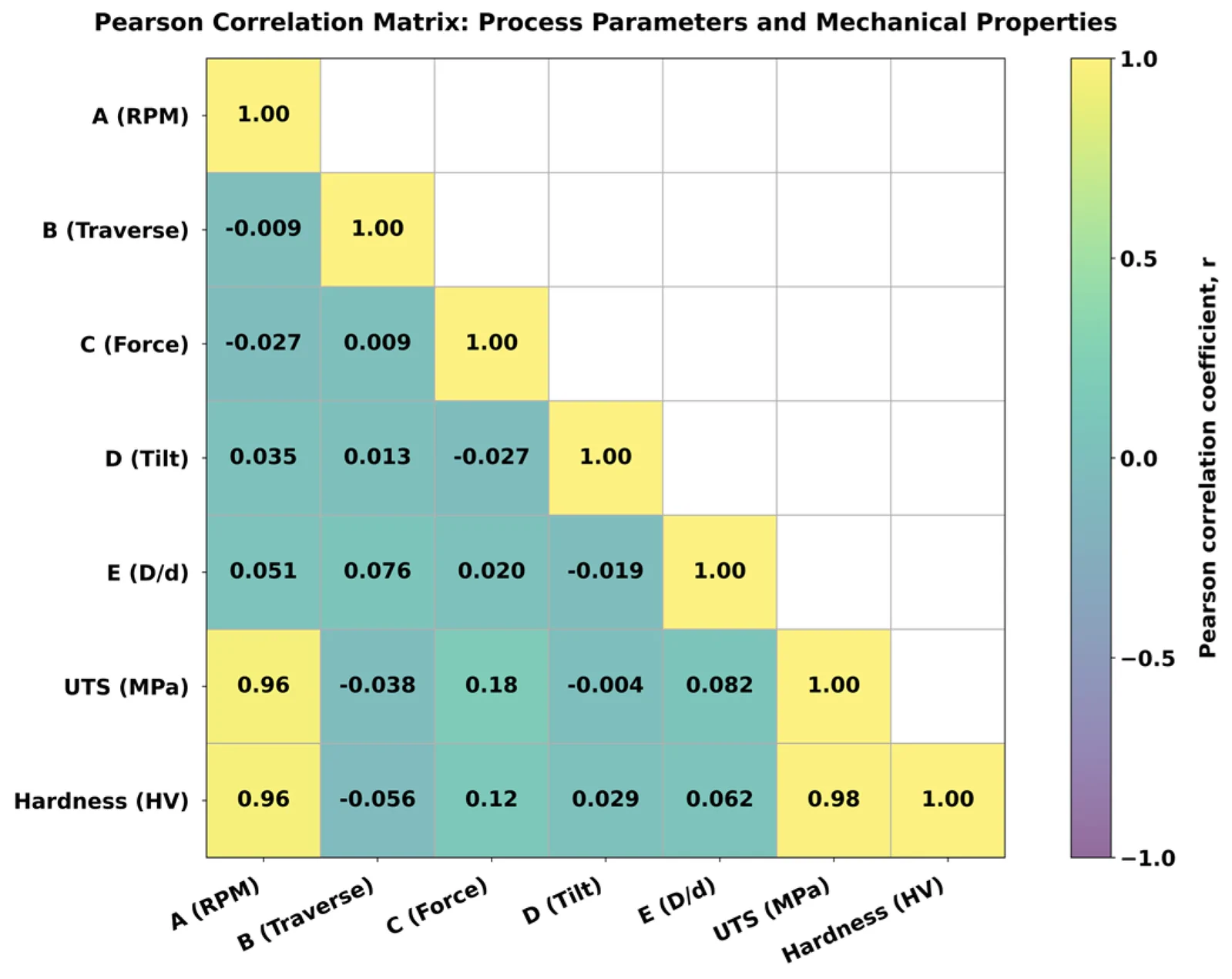
Pearson correlation coefficient matrix.

Existing process-parameter optimization has expanded from single-factor experiments to response surface methodology, the Taguchi method, and machine learning, but most models remain limited to a small number of responses, such as hardness, strength, or wear behavior. Response surface models can predict the strength and hardness of AA7075/ZrO_2_/graphene composites with relatively high accuracy [44]. Although the random forest, gradient boosting, and extremely randomized tree models exhibit high fitting accuracy, the Pearson correlation coefficient matrix shown in Figure 7 can be used to characterize the degree of linear correlation between individual process parameters and tensile strength or hardness, thereby assisting in the identification of variables to which the performance responses are more sensitive. However, correlation does not imply causation, and the model training data remain limited to the parameter space covered by the L16 orthogonal experimental design [77]. Coupled temperature-field–grain–precipitate models can be used to explain the effects of particle size and volume fraction [78], while in-situ force and torque signals can characterize material-flow conditions, equipment loading, and the risk of tool failure [62]. Yasa et al. [80] simultaneously acquired temperature, force, vibration, and acoustic signals during AFSD of Al6061 and compared the process responses under PID-controlled and non-PID conditions, demonstrating that multisource process information can capture state fluctuations across different deposition stages. Abir et al. [81] further developed a Wasserstein-distance-based process dissimilarity index using machine sensor outputs, which enables quantitative assessment of process consistency among different builds and deposited layers, identification of process deviations caused by parameter changes and manufacturing interruptions, and correlation of these deviations with variations in material hardness. Kumar et al. [82] noted that the application of machine learning in AFSD is evolving from offline parameter optimization toward real-time defect detection, adaptive process adjustment, and digital process integration.

Therefore, process-parameter control should shift from empirical open-loop adjustment toward closed-loop quality control based on multisource information such as temperature, force, torque, and acoustic emission [9]. For internal quality assessment, the integration of machine learning with industrial X-ray computed tomography (XCT) has been applied to image reconstruction, segmentation, and feature characterization [83], providing methodological support for the quantitative analysis of porosity, interlayer interfaces, and the three-dimensional distribution of reinforcement phases in AFSD deposits, while also serving as a quasi-in-situ or offline reference for correlating online process signals with internal quality. Current effective control strategies include using premixed or prefabricated composite feedstocks to reduce initial agglomeration; employing helical, eccentric, or polygonal tools to enhance vertical material flow; appropriately limiting the content of high-hardness particles; increasing interlayer replasticization through pin penetration depth, overlap width, and axial load; suppressing build-height gradients through interlayer cooling, thermally balanced deposition paths, or localized heat treatment; and applying hybrid additive–subtractive finishing to edge flash and weakly bonded regions. The optimization objectives should simultaneously cover forming integrity, particle distribution, interfacial strength, mechanical properties in three directions, and tool loading, with coordinated design centered on reinforcement dispersion and interlayer integrity [14].

Beyond composite materials, AFSD studies on unreinforced matrix materials provide an essential baseline for identifying the “incremental effects” associated with reinforcement introduction. Studies on AA7050 and AA5083 have revealed thermal accumulation, precipitate evolution, lubricant contamination, and build-direction-dependent property variations [84,85], while investigations of thin-sheet cladding and direct chip recycling have demonstrated the application boundaries of AFSD for low-residual-stress localized strengthening and resource recycling [86,87]. Deposition studies of WE43 and AZ31B magnesium alloys and metastable high-entropy alloys further indicate that dynamic recrystallization mechanisms, texture/phase-transformation behavior, and acceptable processing windows strongly depend on the constitutive response and thermophysical properties of the matrix material [88,89,90]. These cross-material baselines indicate that the processing windows of composites should be modeled incrementally by considering the “thermomechanical response of the matrix + reinforcement-induced changes in viscosity, friction, and interfacial behavior,” rather than by directly applying empirical parameters established for a single aluminum alloy.

Research on underlying mechanisms, environmental boundaries, and process control is also continuing to advance. Coupled experimental–computational studies of temperature and strain rate have quantified the influence of constitutive models on the mechanical response of AFSD [91]; underwater deposition of 304 stainless steel has verified the feasibility of solid-state deposition under forced-cooling conditions [92], while studies on SS316 have revealed the contributions of discontinuous dynamic recrystallization, twinning, and phase transformation to the strength–ductility response of low-stacking-fault-energy materials [93]. Furthermore, the three-dimensional flow trajectories and extreme thermomechanical histories of material voxels can now be quantitatively tracked using experimentally constrained models [94]; closed-loop control based on tool-temperature feedback has been implemented for Ti-6Al-4V, achieving transverse tensile properties meeting forging standards [95], and the tensile and fatigue performance of directly additively recycled AA5083 chips has also been validated [96]. Interpretable thermal-field prediction enhanced by Bayesian learning [97], together with forging-grade tensile properties achieved in AA7050 through tailored heat treatment [98], indicates that AFSD is evolving from “manufacturability” toward “predictability, controllability, and certifiability”; for composite materials, the next step should be to incorporate reinforcement concentration, three-dimensional distribution, interfacial state, and tool loading into a unified closed-loop evaluation framework.

To distinguish the effects of solid-state friction-based additive manufacturing from those associated with the initial material condition and conventional processing routes, representative studies are compared in Table 6. In conventional aluminum-matrix composites, Ti coatings can improve fatigue life and fatigue limit, with these benefits retained at 200 °C [99]. After friction stir additive manufacturing, AA6061-T6 exhibits increased elongation and impact energy but reduced strength and hardness [100]; by contrast, Al–12Ce–3Mg–0.45Sc transforms from a nearly brittle as-cast condition to a high-strength and high-toughness state after AFSD [101]. Following heat treatment, 6061–7075 composite AFSD material can recover properties approaching those of forged AA6061 [102], whereas Al5086-H32 exhibits reduced strength and hardness but increased elongation [103]. In addition, the fatigue life of Al7075-T6 decreases with increasing deposition height [104], whereas Al–Cu–Mg–Ag can achieve properties close to those of conventionally hot-extruded and T6-treated material through optimization of the initial condition [105]. Overall, mechanical properties are jointly governed by the initial material condition, thermal history, recrystallization/precipitation evolution, and post-processing, rather than being determined solely by the deposition route.

Solid-state friction-based additive manufacturing does not exhibit a single, universal pattern of performance improvement relative to conventional manufacturing processes. For as-cast materials or alloys containing coarse secondary phases, severe shearing, dynamic recrystallization, and particle fragmentation can simultaneously improve strength and ductility; however, for alloys initially in strengthened conditions such as T6 or H32, deposition-induced thermal cycling may weaken precipitation strengthening or strain-hardening effects, resulting in reduced strength and hardness but increased ductility. Impact performance is governed by interlayer bonding, crack deflection, and plastic energy dissipation, whereas fatigue performance is more sensitive to surface/internal defects, precipitate stability, and the thermal history along the build-height direction. Therefore, practical engineering assessment should rely on paired data obtained under identical material compositions, heat-treatment conditions, sampling orientations, and testing conditions, rather than ranking different material systems solely on the basis of absolute strength or hardness values reported in separate studies.

## 6. Discussion

Based on the available studies, the performance enhancement of composites fabricated by friction stir deposition does not arise solely from the incorporation of reinforcement phases, but rather depends on the synergistic effects of reinforcement dispersion, interfacial bonding, and interlayer consolidation. Moderate heat input and sufficient plastic flow promote dynamic recrystallization, particle redistribution, and interfacial bonding, whereas excessive heat input may cause precipitate dissolution, particle agglomeration, and microstructural gradients; an excessive reinforcement content can also increase material-flow resistance and tool loading. Therefore, the “optimal parameters” identified for different material systems are strongly dependent on the material, tool geometry, and thermal history and cannot be directly transferred across systems.

Another limitation of the existing literature is the lack of standardized evaluation methods. Most studies focus on hardness, room-temperature tensile properties, and localized wear behavior, whereas fatigue, impact, creep, and coupled corrosion–mechanical loading performance remain insufficiently investigated; meanwhile, differences in feedstock condition, sampling orientation, post-processing heat treatment, and component scale also restrict direct comparisons among studies. Therefore, future evaluations should move beyond single performance metrics toward comprehensive characterization of forming quality, interfacial integrity, anisotropy, and in-service performance, while strengthening comparisons with compositionally equivalent materials produced by conventional routes such as casting, forging, and extrusion. From the perspective of engineering applications, stable composite feedstock delivery, quantitative control of reinforcement content, tool life, and microstructural consistency along the build height remain major bottlenecks. Multisource process information, three-dimensional defect characterization, and data-driven models can improve the capabilities for abnormal-state identification and parameter adjustment, but their applicability across different materials and equipment still requires validation through large-scale component fabrication and long-term service testing.

## 7. Conclusions and Outlook

Friction-stir-deposition-based solid-state additive manufacturing can be used to fabricate metal matrix composites reinforced with particles, nanosheets, short fibers, continuous meshes, functional gradients, and in-situ reaction products. These processes induce severe plastic deformation through thermomechanical coupling, enabling densification, reinforcement redispersion, and interlayer metallurgical bonding without bulk melting of the matrix. At present, aluminum-matrix composites have been mostly investigated systematically, and rods, powder mixtures, preformed grooves or holes, composite wires, and predeposited layers can all serve as carriers for reinforcement introduction.

Microstructural and property control in composites mainly relies on mechanisms including dynamic recrystallization, grain refinement, grain-boundary and dislocation pinning, Orowan strengthening, and load transfer, while the reinforcing phases can also regulate precipitation behavior, form interfacial transition layers, and impart wear resistance, corrosion resistance, and radiation-shielding functions. However, the strengthening effect depends on the synergistic interaction among reinforcement dispersion, interfacial bonding, and interlayer consolidation; excessive reinforcement content, inappropriate size matching, or excessive heat input can induce agglomeration, interfacial debonding, precipitate dissolution, and tool wear, thereby degrading the overall properties.

The effects of processing parameters on material flow, heat input, and strain rate exhibit a pronounced dependence on the material system. Complex interactions exist among rotational speed, feed rate, axial load, tool tilt angle, shoulder-to-pin size ratio, number of deposition passes, and overlap ratio, making it difficult to establish universally optimal parameters across different material systems. Future studies should establish a multi-criteria evaluation framework covering geometric accuracy, interlayer bonding, reinforcement distribution, microstructural gradients, and anisotropic mechanical properties, rather than using local hardness to represent the overall quality of a component. Meanwhile, feedstock design should evolve from simply being “introducible” toward quantitative, continuous, and traceable control, with emphasis on compositional calibration of composite rods and wires, segregation-resistant continuous feeding, and three-dimensional quantitative characterization of reinforcement volume fraction.

Future research should strengthen investigations into both process mechanisms and engineering applications by synchronously acquiring temperature, load, torque, and material-flow information and combining these data with in-situ or quasi-in-situ three-dimensional characterization to reveal the mechanisms of reinforcement fragmentation, migration, agglomeration, and interfacial evolution; micropillar compression, nanoindentation, interfacial shear, and fracture tests should be used to quantify interfacial strength and load-transfer efficiency, together with the development of coupled thermal–mechanical–flow multiphysics models. For engineering implementation involving large-scale components, complex deposition paths, and long-term service conditions, real-time quality assurance should integrate multisource signals such as temperature, load, torque, vibration, and acoustic emission (AE), together with machine learning and physics-constrained models for abnormal-state identification and adaptive closed-loop regulation of rotational speed, feed rate, and axial load; XCT can serve as a quasi-in-situ/offline three-dimensional quality benchmark and be correlated with online signals to improve the reliability of internal defect identification and quality traceability. Sampling protocols across different layers and orientations should be standardized, and databases covering fatigue, creep, coupled corrosion–mechanical loading, and wear life should be further developed. Only through systematic quantification of deposition efficiency, material utilization, tool life, post-processing allowance, and batch-to-batch consistency can this technology progress from laboratory-scale specimen fabrication toward the manufacturing of highly reliable, reproducible, and certifiable components.

In summary, the main contribution of this review is the establishment of a cross-process comparative framework for solid-state friction-based additive manufacturing of composites, centered on controllable reinforcement incorporation, material flow and interfacial evolution, microstructural defects, and property responses, while systematically integrating the process–microstructure–property evidence and engineering bottlenecks dispersed across different material systems. This framework can provide systematic guidance for material and parameter selection, quality assessment, and scale-up manufacturing of composite systems.

## Figures and Tables

**Table 1:** Principal material systems investigated for composite additive friction stir deposition.

Matrix Material	Reinforcement	Representative System	Microstructural/Performance	Challenge	Ref.
6xxx aluminum alloys	SiC particles	SiC_p_/6061	Grain refinement; increased strength and hardness	Agglomeration at high reinforcement contents; reduced elongation	[50]
6xxx aluminum alloys	ZrO_2_ particles	AA6061–ZrO_2_	Improved hardness, strength, and wear resistance	Control of reinforcement content and feedstock consistency	[62]
6xxx aluminum alloys	CNTs	CNT/AA6061	Fine grains; high strength and ductility; reduced corrosion current	Agglomeration and inconsistent trends among corrosion indicators	[55]
6xxx aluminum alloys	GNPs	GNP/AA6061	Strength improvement after exceeding a threshold reinforcement content	Insufficient strengthening at low contents; high sensitivity to dispersion	[35]
2xxx aluminum alloys	Nano-Al_2_O_3_	AA2011/Al_2_O_3_	Improved hardness, compressive performance, and wear resistance	Strong influence of the initial heat-treatment condition	[63]
2xxx aluminum alloys	CNTs	CNT/2A12	Fine grains; high strength and ductility; reduced corrosion current	Insufficient long-term corrosion and fatigue data	[56]
5xxx aluminum alloys	TiO_2_ + CeO_2_	AA5083/TiO_2_–CeO_2_	Tunable hardness, wear resistance, and passivation behavior	Agglomeration at high CeO_2_ contents	[64]
5xxx aluminum alloys	MoS_2_ + nanodiamond	AA5083-H321/(MoS_2_ + ND)	Combined regulation of hardness, wear, and corrosion performance	Changes in rotational speed and feed rate can alter particle segregation	[60]
7xxx aluminum alloys	SiC or TiC	Al7075/SiC; Al7075/TiC	TiC systems show finer grains and more favorable tool-wear behavior	High SiC contents cause material-flow restriction	[53]
Dissimilar Al/Mg laminated metal matrix	Graphene	Al2024/AZ31B/graphene	Improved interfacial hardness and tribological performance	Dissimilar-interface reactions and sensitivity to the processing window	[65]
6xxx aluminum alloys	Basalt fibers	BF/AA6061	Strength–ductility balance and crack deflection	Control of fiber fragmentation and size distribution	[57]
Pure Al	Steel mesh	Steel-mesh-reinforced Al-matrix composite	Enhanced directional load-bearing capability with retained ductility	Long-term interfacial stability remains to be validated	[38]
6xxx aluminum alloys	Lunar regolith simulant	AA6061/lunar regolith simulant	High build height with low porosity can be achieved	Ductility loss and build-direction variability	[66]
Pure Al	MgB_2_	Al/MgB_2_	Integration of wear resistance and proton-shielding functionality	Insufficient data under space-service conditions	[67]
Al-based energetic materials	Ni, CuO	Al/Ni/CuO	Coupled variation in strength and exothermic energy density	Insufficient assessment of reaction safety and thermal stability	[58]

**Table 2:** Typical reinforcement introduction methods and their process characteristics.

Introduction Method	Representative Feedstock	Forming Characteristics	Major Risks	Ref.
Central filling of hollow rods	Al6061–B_4_C	Continuous feeding; suitable for low-to-moderate particle contents	Axial concentration fluctuations and localized agglomeration	[32]
Powder filling of predrilled/porous rods	AA2014–SiC	Particle volume fraction can be preset; relatively strong interfacial bonding	Filling errors and feed interruption	[68]
Powder-blend tube filling	AA6061–SiO_2_	Adjustable composition ratio; particle morphology can be used to trace material flow	Unmixed regions near the tube wall and radial nonuniformity	[34]
Commercial composite feedstock	Al–SiC	Relatively stable initial particle distribution; suitable for continuous deposition	Substrate dilution and inherited particle orientation	[36]
Powder-metallurgy/extruded composite rods	Al7075-equivalent matrix/Al_2_O_3_	Porosity can be compensated through feed-rate adjustment	Relatively lengthy feedstock-preparation route	[33]
Preplacement in sheet grooves or holes	AA7075–ZrO_2_–Gr	Flexible composition control; suitable for laminated and graded designs	Incomplete closure and residual grooves	[44]
Pass-by-pass stirring of powder layers	Al6061–FeCoNi	Facilitates heterogeneous powder combinations and adjustment of interlayer rotation direction	Powder loss and surface accumulation	[37]
Continuous composite wire	TiC/Al–Cu–Mg	Continuous feeding; pre-plasticization promotes fragmentation of agglomerates	Wire consistency and screw service life	[39]
Predeposited fibers + hollow rod	BF/AA6061	Fibers can be entrained into the deposit and fragmented into dual-scale constituents	Excessive fiber fragmentation or segregation	[57]
Continuous mesh embedding	Steel mesh/Al	Retains the predefined orientation and enables directional load bearing	Stringent requirements for interfacial encapsulation and path alignment	[38]

**Table 3:** Typical defects, their formation conditions, identifying characteristics, and suppression strategies.

Defect/ Heterogeneity	Formation Conditions	Typical Characteristics	Effects	Feasible Mitigation Strategies	Ref.
Particle agglomeration	Excessive reinforcement content, insufficient feedstock mixing, or segregation at high rotational speed	Localized particle-rich regions, voids, and interfacial debonding	Reduced ductility and crack initiation	Reduce reinforcement content, improve premixing, and optimize vertical material flow	[50]
Lamellar porosity/unbonded regions	Insufficient heat input or inadequate material flow	Continuous interlayer porosity and reduced properties along the build direction	Anisotropy and premature fracture	Increase rotational speed, pin penetration depth or axial consolidation	[12]
Tunnel defects	Flow restriction caused by excessive reinforcement content	Internal voids extending along the traverse direction	Reduced strength and increased tool loading	Optimize reinforcement volume fraction and tool geometry	[49]
Overlap kissing-bond defects	Insufficient overlap width and inadequate repeated plasticization	No obvious open gap at the interface, but weak bonding persists	Reduced shear and tensile performance	Increase overlap width and apply secondary processing	[47]
Weak edge bonding	Reduced thermomechanical action near the lateral edges	Edge shear strength markedly lower than that in the central region	Risk of surface-layer delamination	Remove weak edge regions or apply secondary stirring/impact treatment	[48]
Initial-layer/substrate-interface porosity	Unstable material flow during initial deposition and sidewall flash formation	Porosity concentrated in the first layer and outer-edge regions	Reduced effective build width	Optimize the initial deposition path and machine the outer edges	[66]
Tool wear or fracture	High-hardness/high-content particles and high material-flow resistance	Reduced pin diameter, loss of tool features, or pin fracture	Build interruption and compositional contamination	Use wear-resistant tools or coatings and control reinforcement content	[53]
Property gradients	More extensive thermal cycling near the bottom, precipitate coarsening, or grain growth	Hardness and strength vary along the build height	Reduced quality consistency	Apply interlayer cooling, thermally balanced processing, and post-deposition heat treatment	[73]

**Table 4:** Effects of the main process parameters on heat input, material flow, and forming quality.

Parameter	Primary Process Response Induced by Variation	Potential Improvement	Potential Deterioration	Applicability/Boundary Condition	Ref.
Tool rotational speed	Changes frictional heat generation, shear frequency, and degree of plasticization	Enhances material flow, recrystallization, and interlayer bonding	Excessive speed may cause grain coarsening, precipitate dissolution, and reinforcement segregation	AA6061/TiC–Gr exhibits a performance trade-off within 1200–1500 r/min	[76]
Material feed rate	Changes the amount of feedstock supplied per unit time and the axial pressure	A moderate increase can improve deposition efficiency and consolidation	Excessive feeding may cause overfeeding, increased thermal gradients, and more flash	AA5083/MoS_2_–diamond system	[60]
Tool traverse speed	Changes heat input per unit length and material residence time	A relatively low speed can enhance mixing and bonding	Too low a speed may cause heat accumulation, whereas too high a speed may lead to insufficient plasticization	Optimized for AA7075/Gr–B_4_C	[77]
Axial load	Increases contact pressure, interfacial filling, and vertical material flow	Suppresses porosity and improves interlayer contact	Excessive load may cause material overflow and increased tool loading	Taguchi analysis indicates a coupled effect with rotational speed	[77]
Tool tilt angle	Regulates trailing-edge forging pressure and material backfilling	Improves trailing-edge closure and reduces groove formation	Excessive tilt may cause surface pile-up or thickness fluctuations	Optimized to 3° for AA7075/ZrO_2_/Gr	[44]
Pin penetration depth/layer height	Determines the replasticization depth of the preceding layer	Enhances cross-layer mixing and build-direction properties	Excessive penetration may damage the substrate or disturb reinforcement distribution	SiC/AA6061 employs a pin penetration of 50% of the layer height	[73]
Overlap width	Changes the repeatedly forged zone and filled volume	Increasing overlap can eliminate overlap porosity and kissing-bond defects	Excessive overlap reduces deposition efficiency and increases heat accumulation	A 5 mm overlap provides relatively high interfacial strength	[47]
Number of passes	Increases repeated stirring, particle fragmentation, and defect closure	Improves reinforcement dispersion and refines the microstructure	Heat accumulation may cause grain coarsening and hardness reduction	Functionally graded Al/TiC exhibits a trade-off with the number of passes	[69]

**Table 5:** Performance results and improvement trends of representative composite systems.

Material System	Process/Key Condition	Main Performance Results	Sources of Performance Improvement and Trade-Offs	Ref.
TiC/AA6061	FEAM; post-deposition heat treatment	As-deposited: 76.5 HV and 244.2 MPa; after heat treatment: 114.6 HV and 313 MPa	Particle strengthening and recovery through heat treatment; localized interfacial defects remain	[10]
AA2014/SiC	AFD; porous-rod feedstock	240 ± 5 HV; interfacial shear strength of 140 ± 5 MPa; wear loss reduced by 36%	Fine-grain, particle, and dislocation strengthening; the heat-treatable matrix may undergo overaging	[68]
ZrB_2_/6061	6 vol.% ZrB_2_	Hardness increased by 35.62%; strength increased by 14.2%; corrosion current density decreased by 87.1%	Combined grain refinement and precipitate reconstruction; agglomeration occurs at 8 vol.%	[75]
SiC_p_/6061	15% SiC	83.75 HV; tensile strength of 140.15 MPa; elongation of 18.59%	Strength and hardness increase, whereas ductility decreases at high reinforcement contents	[50]
CNT/2A12	AFSD; 22 mm-high build	Tensile strength > 450 MPa; elongation ≥ 15%; corrosion current density of 0.19 μA/cm^2^	Grain refinement and homogenization of precipitates; insufficient long-term corrosion data	[56]
Dual-scale BF/AA6061	Predeposited layer + hollow rod	Yield strength of 139 MPa; tensile strength of 287 MPa; elongation of 27%	Dislocation strengthening, Orowan strengthening, crack deflection, and fiber pull-out	[57]
TiB_2_/7075	W-FSAM; eccentric single pin	Tensile strength of 391–409 MPa in three directions; elongation of 12.4–13.4%	Vertical mixing and grain refinement produce near-isotropic properties	[31]
Nano-Al_2_O_3_/ 2009Al	AFED; 600 r/min	Tensile strength of 675–695 MPa in different directions; elongation of 6–7%	Interlayer defects are eliminated, although ductility remains limited by the high-strength matrix	[12]
Al/MgB_2_	Stacked FSAM; high particle content	Hardness of approximately 191 HV; proton-stopping capability higher than that of pure Al/Mg	Integration of structural and radiation-shielding functions; insufficient service-condition validation	[67]
Steel mesh/Al	12.3 vol.% steel mesh	Tensile strength increased by 27.1%; flexural strength increased by 41.8%; no loss of ductility	Continuous reinforcement provides load bearing; long-term interfacial stability remains to be validated	[38]

**Table 6:** Comparison of typical mechanical properties between solid-state friction-based additive manufacturing and conventional processing conditions.

Materials	Tensile Properties	Hardness	Impact/Fatigue Properties	Ref.
Al6061, A359, and 2618/Al_2_O_3_ or SiC MMCs; conventionally fabricated by fusion processing/powder metallurgy, with comparison before and after Ti coating	—	—	Ti coating can significantly improve fatigue life and endurance limit, with improvements also observed at 200 °C.	[99]
AA6061-T6 base plate vs. 15-layer FSAF component	The base material exhibited a UTS of 310 MPa and an elongation of 18%, whereas the FSAF material showed a UTS of approximately 224.71–252.60 MPa and an increased elongation of 50.18–66%.	The hardness decreased from 108 HV to a maximum of 82.48 HV, with approximately 58.91 HV measured at the bottom.	The impact energy increased from 18 J in the base material to a maximum of 112 J in the crack-arrest direction and 86 J in the crack-divider direction.	[100]
Squeeze-cast Al–12Ce–3Mg–0.45Sc vs. AFSD	The as-cast material fractured prematurely at approximately 230 MPa with almost no ductility, whereas the AFSD material achieved a YS of 385 MPa, a UTS of 530 MPa, and a plastic strain of 12.6%.	Hardness increased from 119 ± 4 HV to 128 ± 3.8 HV.	—	[101]
061–7075 composite AFSD + heat treatment vs. forged AA6061 engineering benchmark	AFSD + HT: YS of 289 MPa, UTS of 368 MPa, and elongation of 22%, which were reported to be comparable to those of forged AA6061.	115 HV_0_._5._	—	[102]
Al5086-H32 feedstock rod vs. AFSD component	decreased from 195 to 150 MPa, UTS decreased from 303 to 290 MPa, while elongation increased from 20% to approximately 36%.	Hardness decreased from 91 HV to 88.8 HV at the top and 76 HV at the bottom.	—	[103]
Al7075-T6 feedstock rod vs. top/bottom regions of high-speed AFSD build	—	—	The bending fatigue life decreased from 221,000 cycles to 174,000 cycles at the top and 164,000 cycles at the bottom.	[104]
Conventional hot extrusion + T6 Al–Cu–Mg–Ag vs. AFSD with different feedstock conditions	Conventional hot extrusion + T6: UTS of 400 MPa and elongation of 12.3%; AC-ST-AFSD: UTS of 412 MPa and elongation of 12.1%.	—	—	[105]

## Data Availability

No new data were created or analyzed in this study. Data sharing is not applicable to this article.

## Nomenclature

Acronym	Full Term
AFSD	Additive Friction Stir Deposition
FSAM	Friction Stir Additive Manufacturing
FEAM	Friction Extrusion Additive Manufacturing
AFED	Additive Friction Extrusion Deposition
W-FSAM	Wire-Based Friction Stir Additive Manufacturing
IFSAR	In-situ Friction Stir Additive Repair
AFD	Additive Friction Deposition
CNT	Carbon Nanotube
GNP	Graphene Nanoplatelet
Gr	Graphene
BF	Basalt Fiber
EBSD	Electron Backscatter Diffraction
TEM	Transmission Electron Microscopy
XCT	X-ray Computed Tomography
PCD	Polycrystalline Diamond
UTS	Ultimate Tensile Strength
HV	Vickers Hardness

## References

[1] Li Y., Li X., He C., Li Y., Wen K., Yan L., Xiao W., Zhang Y., Xiong B., Montealegre-Meléndez I. (2024). A review of solid-state additive manufacturing: Method, microstructural evolution, mechanical properties, applications and challenges. Additive Manufacturing—Present and Sustainable Future, Materials and Applications.

[2] Dong H., Li X., Xu K., Zang Z., Liu X., Zhang Z., Xiao W., Li Y. (2022). A review on solid-state-based additive friction stir deposition. Aerospace.

[3] Li X., Chen Y., Chen H., Jun X. (2025). Research progress in regulation of residual stress and distortion in wire arc additive manufacturing. J. Mater. Eng..

[4] Treutler K., Wesling V. (2025). New developments in the field of production and application of multi-material wire arc additive manufacturing components: A review. Adv. Eng. Mater..

[5] Falco R., Bagherifard S. (2025). Cold spray additive manufacturing: A review of shape control challenges and solutions. J. Therm. Spray Technol..

[6] Badaloo A., Faizan-Ur-Rab M., Zahiri S.H., Masood S.H., Palanisamy S. (2025). Assessment of current capabilities for cost effective digital deposition of cold spray additive structures: A review. Int. J. Adv. Manuf. Technol..

[7] Gopan V., Leo Dev Wins K., Surendran A. (2021). Innovative potential of additive friction stir deposition among current laser based metal additive manufacturing processes: A review. CIRP J. Manuf. Sci. Technol..

[8] Li X., Li X., Hu S., Liu Y., Ma D. (2024). Additive friction stir deposition: A review on processes, parameters, characteristics, and applications. Int. J. Adv. Manuf. Technol..

[9] Yasa E., Poyraz O., Molyneux A., Sharman A., Bilgin G.M., Hughes J. (2024). Systematic review on additive friction stir deposition: Materials, processes, monitoring and modelling. Inventions.

[10] Dong Q., Liu X., Yan S., Ren X. (2025). A new method to fabricate TiC reinforced *AA*6061 aluminum matrix composites using friction extrusion additive manufacturing. SSRN.

[11] Wei S.Z., Liu F.C., Wang Y.D., Ma X., Liu Z.Y., Yang J.X., Xue P., Zhang Z., Wu L.H., Zhang H. (2025). Additive manufacturing of heat-treatment-free high-strength CNT/Al6Mg nanocomposites using emerging additive friction extrusion deposition. Adv. Compos. Hybrid Mater..

[12] Zhou X.L., Liu Z.Y., Liu F.C., Song K.P., Zeng H., Ma K., Xiao B.L., Ma Z.Y. (2026). Eliminating interlayer defects for achieving isotropic and superior mechanical properties in additive friction extrusion deposited nano-Al_2_O_3_/2009Al composites. Mater. Charact..

[13] Li Z., Chen S., Xiao J., Lu X., Chen S., Gai S. (2025). Strengthening and toughening mechanisms in in-situ friction stir additive repair (IFSAR) of volume defects in 2195 aluminum-lithium alloys reinforced by TiC particles. J. Manuf. Process..

[14] Magudu A., Msomi V., Mabuwa S. (2026). Recent advances in friction stir additive manufacturing: A review of mechanical and microstructural performance in similar and dissimilar composites. Mechanika.

[15] Verma R., Kumar A., Shrivastava P., Sharma A. Fabrication and characterization of metal matrix composite by friction stir additive manufacturing (FSAM): A review. Proceedings of the International Conference on Science, Engineering and Technology (ICSET 2022).

[16] Yu H.Z., Jones M.E., Brady G.W., Griffiths R.J., Garcia D., Rauch H.A., Cox C.D., Hardwick N. (2018). Non-beam-based metal additive manufacturing enabled by additive friction stir deposition. Scr. Mater..

[17] Khodabakhshi F., Gerlich A.P. (2018). Potentials and strategies of solid-state additive friction-stir manufacturing technology: A critical review. J. Manuf. Process..

[18] Griffiths R.J., Perry M.E.J., Sietins J.M., Zhu Y., Hardwick N., Cox C.D., Rauch H.A., Yu H.Z. (2019). A perspective on solid-state additive manufacturing of aluminum matrix composites using MELD. J. Mater. Eng. Perform..

[19] Griffiths R.J., Petersen D.T., Garcia D., Yu H.Z. (2019). Additive friction stir-enabled solid-state additive manufacturing for the repair of 7075 aluminum alloy. Appl. Sci..

[20] Phillips B.J., Avery D.Z., Liu T., Rodriguez O., Mason C., Jordon J., Brewer L., Allison P. (2019). Microstructure-deformation relationship of additive friction stir-deposition Al-Mg-Si. Materialia.

[21] Rivera O.G., Allison P.G., Brewer L.N., Rodriguez O., Jordon J., Liu T., Whittington W., Martens R., McClelland Z., Mason C. (2018). Influence of texture and grain refinement on the mechanical behavior of AA2219 fabricated by high shear solid state material deposition. Mater. Sci. Eng. A.

[22] Rivera O.G., Allison P.G., Jordon J.B., Rodriguez O., Brewer L., McClelland Z., Whittington W., Francis D., Su J., Martens R. (2017). Microstructures and mechanical behavior of Inconel 625 fabricated by solid-state additive manufacturing. Mater. Sci. Eng. A.

[23] Avery D.Z., Rivera O.G., Mason C.J.T., Phillips B.J., Jordon J.B., Su J., Hardwick N., Allison P.G. (2018). Fatigue behavior of solid-state additive manufactured Inconel 625. JOM.

[24] Avery D.Z., Phillips B.J., Mason C.J.T., Palermo M., Williams M.B., Cleek C., Rodriguez O.L., Allison P.G., Jordon J.B. (2020). Influence of grain refinement and microstructure on fatigue behavior for solid-state additively manufactured Al-Zn-Mg-Cu alloy. Metall. Mater. Trans. A.

[25] Garcia D., Hartley W.D., Rauch H.A., Griffiths R.J., Wang R., Kong Z.J., Zhu Y., Yu H.Z. (2020). In situ investigation into temperature evolution and heat generation during additive friction stir deposition: A comparative study of Cu and Al-Mg-Si. Addit. Manuf..

[26] Perry M.E.J., Griffiths R.J., Garcia D., Sietins J.M., Zhu Y., Yu H.Z. (2020). Morphological and microstructural investigation of the non-planar interface formed in solid-state metal additive manufacturing by additive friction stir deposition. Addit. Manuf..

[27] Yu H.Z., Mishra R.S. (2021). Additive friction stir deposition: A deformation processing route to metal additive manufacturing. Mater. Res. Lett..

[28] Griffiths R.J., Garcia D., Song J., Vasudevan V.K., Steiner M.A., Cai W., Yu H.Z. (2021). Solid-state additive manufacturing of aluminum and copper using additive friction stir deposition: Process-microstructure linkages. Materialia.

[29] Perry M.E.J., Rauch H.A., Griffiths R.J., Garcia D., Sietins J.M., Zhu Y., Zhu Y., Yu H.Z. (2021). Tracing plastic deformation path and concurrent grain refinement during additive friction stir deposition. Materialia.

[30] Stubblefield G.G., Fraser K., Phillips B.J., Jordon J., Allison P. (2021). A meshfree computational framework for the numerical simulation of the solid-state additive manufacturing process, additive friction stir-deposition (AFS-D). Mater. Des..

[31] Sun S., Meng X., Xie Y., Wang J., Ma X., Wang N., Li X., Huang Y. (2025). Wire-based friction stir additive manufacturing enables enhanced interlayer bonding in aluminum-matrix composites. J. Manuf. Process..

[32] Mani Krishna K.V., Patil S.M., Sharma S., Joshi S.S., Jin Y., Radhakrishnan M., Dahotre N.B. (2024). Additive friction stir deposition of Al 6061-B4C composites: Process parameters, microstructure and property correlation. Mater. Sci. Eng. A.

[33] Ding H., Emanet S., Zavari S., Thai T.D., Chen Y., Bagheri E., Guo S. (2025). In-situ aluminum 7075 metal matrix composites development for additive friction stir deposition. J. Mater. Res. Technol..

[34] Lopez J.J., Williams M.B., Rushing T.W., Jordon J.B., Allison P.G., Thompson G.B. (2024). Silica particulate dispersion during additive friction deposition in a metal matrix composite. J. Mater. Res. Technol..

[35] Lopez J.J., Williams M.B., Rushing T.W., Bikmukhametov I., Jordon J.B., Allison P.G., Thompson G.B. (2024). Solid-state additive manufacturing of dispersion strengthened aluminum with graphene nanoplatelets. Mater. Sci. Eng. A.

[36] Pack R.C., Bohling J.W., Kincaid J., Abir A.I., Schmitz T.L., Compton B.G. (2025). Additive friction stir deposition of multi-layer aluminum-silicon carbide metal matrix composites. Addit. Manuf. Lett..

[37] Chaudhary B., Patel M., Jain N.K., Murugesan J., Patel V. (2023). Friction stir powder additive manufacturing of Al 6061/FeCoNi and Al 6061/Ni metal matrix composites: Reinforcement distribution, microstructure, residual stresses, and mechanical properties. J. Mater. Process. Technol..

[38] Zhang Z., Wan L., Xie Y., Wen Q., Chen S., Huang Y. (2025). Steel grid-reinforced aluminum matrix composite prepared via wire-based friction stir additive manufacturing. Virtual Phys. Prototyp..

[39] Zhang Z., Wan L., Wen Q., Shi Y., Feng Z. (2025). Wire-based friction stir additive manufacturing of TiC reinforced Al-Cu-Mg composite: Particle refinement and dispersion. Compos. Part A Appl. Sci. Manuf..

[40] Garcia D., Griffiths R.J., Yu H.Z. Additive friction stir deposition for fabrication of silicon carbide metal matrix composites. Proceedings of the ASME 2020 15th International Manufacturing Science and Engineering Conference (MSEC2020).

[41] Kumar A., Kumar V., Singh R., Rana S.S., Kumar Y., Raja A.R., Maji K. (2026). Tool pin geometry effects in friction stir additive manufacturing of AA7075-O/ZrO_2_/graphene composites. Prog. Addit. Manuf..

[42] Maurya M., Maurya A., Kumar S. (2026). Analysis of tool pin profile shape for three-layer laminated AA6061/TiC/GS composite developed by friction stir additive manufacturing process. J. Mater. Eng. Perform..

[43] Kumar A., Kumar V., Singh R., Rana S.S., Raja A.R., Maji K., Yusufzai M.Z.K., Kumar Y. (2026). Microstructural evolution and particle dispersion in FSAM-processed AA7075/ZrO_2_/Gr composites using a tapered octagonal tool. Prog. Addit. Manuf..

[44] Kumar A., Kumar Y., Maji K., Kumar S. (2026). Optimization of FSAM parameters for tensile strength and hardness of *AA*7075/9.4%ZrO_2_/0.6%Gr composite using RSM technique. Prog. Addit. Manuf..

[45] Sharifizadeh M., Bani Mostafa Arab N., Refahi Oskouei A. (2024). Statistical analysis and optimization of friction stir additive manufacturing process variables for aluminum silicon carbide nanocomposite using the response surface method. Iran. J. Manuf. Eng..

[46] Sharifizadeh M., Bani Mostafa Arab N., Refahi Oskouei A. (2025). The impact of friction stir additive manufacturing parameters on the hardness and wear resistance of aluminum-silicon carbide nanocomposite. Int. J. Interact. Des. Manuf..

[47] He B., Zhang C., Deng C., Yao H., Wang S., Huang Y., Zhu H., Cui L. (2025). Microstructure and properties of the overlap region in SiCp/Al wear-resistant layers on 6061 aluminum alloy fabricated by additive friction stir deposition. Mater. Charact..

[48] He B., Zhang C., Deng C., Yao H., Wang S., Huang Y., Zhu H., Cui L. (2026). Heterogeneity of microstructure and mechanical properties in multi-pass SiCp/Al composite layers fabricated on AA6061 by additive friction stir deposition. J. Alloys Compd..

[49] Hassan A., Altaf K., Che Ismail M., Marode R.V., Soomro I.A., Ahmed N., Awang M. (2026). Fabrication challenges and mechanical properties of SiC-reinforced Al-7075 laminates synthesized via friction stir additive manufacturing: A preliminary study. Prog. Eng. Sci..

[50] Chen T., Chen L., Dong M., Ren X. (2026). Effect of SiC on the microstructure and properties of SiC/Al6061 composites fabricated by additive friction stir deposition. SSRN.

[51] Chen T., Chen L., Dong M., Ren X. (2025). Microstructure and mechanical properties of SiCp/6061 aluminum matrix composites fabricated by additive friction stir deposition. J. Alloys Compd..

[52] Prajapati R., Dwivedi S., Kumar D., Srivastava A.K., Dixit A.R. (2024). Investigation on bonding strength and tribological performances of ceramic laminated AA6063 composite developed by friction stir additive manufacturing. Int. J. Precis. Eng. Manuf.-Green Technol..

[53] Hassan A., Altaf K., Che Ismail M., Pedapati S.R., Marode R.V., Soomro I.A., Ahmed N. (2025). Development of carbide-reinforced Al-7075 multi-layered composites via friction stir additive manufacturing. J. Compos. Sci..

[54] Bagheri E., Adibi N., Ding H., Chen Y., Guo S. (2025). Mechanical and corrosion properties of Al_2_O_3_/7075 aluminum matrix composites prepared by additive friction stir deposition. Prog. Addit. Manuf..

[55] Ghalandari M.A., Mirsalehi S.E., Kiani S. (2025). Production of nanocomposite parts using AA6061-T6 consumable rods via friction stir method: A novel approach of solid-state additive manufacturing of CNT-reinforced aluminum matrix nanocomposites. Mater. Today Commun..

[56] Lei Z., Zhou M., Cao J., Chen G., Xu S., Xue Y., Zhang Y., Shi Q. (2026). Microstructure and properties of CNTs/2A12 aluminum matrix composites fabricated via additive friction stir deposition. Materials.

[57] Wei G., Yang Q., Shen Y., Jia Z., Jiang J., Zhao C., Lyu W., Guo X. (2026). Dual-scale basalt fiber-reinforced AA6061 composites fabricated by additive friction stir deposition. Compos. Part B Eng..

[58] Wang M., Zhu S., Wang T., Ye Y., Wei Z., Wang J., He M., Lu Y., Wang J. (2025). Influence of CuO addition on the microstructure, exothermic characteristics and mechanical properties of Al/Ni/CuO energetic structural materials prepared by friction stir additive manufacturing. J. Mater. Res. Technol..

[59] Kant U., Kumar S., Agrawal A.P., Sharma A. (2022). Characteristics and fabrication of the composite material by the aluminium 6063/B4C/SiC/eggshell through friction stir additive manufacturing. Int. J. Electro Mech. Mech. Behav..

[60] Abbasi-Nahr M., Mirsalehi S.E. (2025). Additive friction stir deposition of AA5083/MoS_2_-diamond hybrid nanocomposites: Investigating their metallurgical, mechanical, tribological, and electrochemical characteristics, and process-structure-property relationships. J. Alloys Compd..

[61] Kumar A., Kumar Y., Maji K. (2025). Advancement and mechanical performance of friction stir additive manufactured Al7075/ZrO_2_/Gr composite. Rapid Prototyp. J..

[62] Qiao Q., Lin Y., Guo D., Kwok C.T., Qian H.C., Li Z., Zhang D., Tam L.M. (2025). Fabrication of an AA6061-ZrO_2_ composite via additive friction stir deposition. J. Alloys Compd..

[63] El-Sayed Seleman M.M., Ataya S., Ahmed M.M.Z., Hassan A.M.M., Latief F.H., Hajlaoui K., El-Nikhaily A.E., Habba M.I.A. (2022). The additive manufacturing of aluminum matrix nano-Al_2_O_3_ composites produced via friction stir deposition using different initial material conditions. Materials.

[64] Navidi Helan M., Mirsalehi S.E. (2024). Additive manufacturing of AA5083/TiO_2_+CeO_2_ hybrid nanocomposites using modified friction stir deposition: Influence of nanoparticles ratio on corrosion resistance, tribological performance, and metallurgical structure evolution. SSRN.

[65] Sharma R., Srivastava A.K., Chaurasia R., Saxena A. (2026). Effect of graphene reinforcement and process parameters on the hardness, tribological, and thermal properties of friction stir additively manufactured Al2024 and AZ31B laminated composite. Can. Metall. Q..

[66] Lopez J.J., Williams M.B., Ravindranath P.K., Rushing T.W., Jordon J.B., Thompson G.B., Allison P.G. (2024). Friction stir additive manufacturing of regolith metal matrix composite. Adv. Space Res..

[67] Srinivasan R., Rajakannu A., Rajesh S., Sathees Babu J., Dinesh G., Mayakannan S. (2025). Friction stir additive manufacturing of AA7075/Al_2_O_3_ and Al/MgB_2_ composites for improved wear and radiation resistance in aerospace applications. J. Environ. Nanotechnol..

[68] Khan M.F., Damodaram R., Patel M.S., Janaki Ram G.D., Karthik G.M. (2026). Development of metal matrix composites through solid-state additive friction deposition. J. Mater. Res. Technol..

[69] Sharma A., Bandari V., Ito K., Kohama K., Ramji M., Sai B.V.H. (2017). A new process for design and manufacture of tailor-made functionally graded composites through friction stir additive manufacturing. J. Manuf. Process..

[70] Jha K.K., Kesharwani R., Mishra R., Imam M. (2024). A clarification on local microstructural inhomogeneity in friction stir additively manufactured functionally graded composite materials. Mater. Today Proc..

[71] He B., Deng C., Yao H., Wang S., Huang Y., Zhu H., Cui L. (2025). Microstructure and mechanical properties of wear-resistant SiCp/Al composite layers on 6061 aluminum alloy fabricated by additive friction stir deposition. Addit. Manuf..

[72] Jha K.K., Kesharwani R., Imam M. (2023). Microstructure, texture, and mechanical properties correlation of AA5083/AA6061/SiC composite fabricated by FSAM process. Mater. Chem. Phys..

[73] Choudhury S., Das R., Sethi D., Roy J., Saha Roy B. (2023). Critical assessment 43: Microstructural and mechanical properties of friction stir additively fabricated SiC-reinforced AA6061 build. Mater. Sci. Technol..

[74] Kiani S., Mirsalehi S.E. (2024). Friction stir additive manufacturing of B4C and graphene reinforced aluminum matrix hybrid nanocomposites using consumable pins. J. Mater. Res. Technol..

[75] Chen Z., Wu Z., Qian H., Wang Z., Qiao Q., Guo D., Zhang D., Kwok C.T., Tam L.M., Li X. (2026). Achieving synergistically enhanced corrosion resistance and hardness in nano-ZrB2/Al composites through friction stir based solid-state additive manufacturing. Corros. Sci..

[76] Sahraei A., Mirsalehi S.E. (2024). An investigation on application of friction stir additive manufacturing (FSAM) for the production of AA6061/TiC-graphene hybrid nanocomposite in the shape of multi-layer cylindrical part. J. Mater. Res. Technol..

[77] Rohan, Sharma A., Ahmad A., Yadav A.K. (2026). Parametric optimization of FSAM-fabricated Al7075/graphene/B4C hybrid composites using a Taguchi-ensemble machine learning framework. Sci. Rep..

[78] Tan Z., Li J., Zhang Z. (2021). Experimental and numerical studies on fabrication of nanoparticle reinforced aluminum matrix composites by friction stir additive manufacturing. J. Mater. Res. Technol..

[79] Razmjoo H., Mirsalehi S.E. (2025). Modified friction stir deposition-additive manufacturing, an approach to fabricate multi-layered AA5083/CeO2/B4C nanocomposites with enhanced corrosion and tribological behavior. Compos. Struct..

[80] Yasa E., Poyraz O., Do K., Molyneux A., McManus J., Hughes J. (2025). In-situ monitoring and control of additive friction stir deposition. Materials.

[81] Abir A.I., Miller L.M., Babu S.S., Compton B.G. (2026). A dissimilarity index for monitoring process consistency in additive friction stir deposition. J. Manuf. Process..

[82] Kumar A., Shi L., Singh V.P., Mohapatra S., Li L., Wu C., Mironov S., De A. (2026). State-of-the-art review of additive friction stir deposition: Microstructural evolution, machine learning applications, and future directions. Curr. Opin. Solid State Mater. Sci..

[83] Bellens S., Guerrero P., Vandewalle P., Dewulf W. (2024). Machine learning in industrial X-ray computed tomography—A review. CIRP J. Manuf. Sci. Technol..

[84] Mason C.J.T., Rodriguez R.I., Avery D.Z., Phillips B., Bernarding B., Williams M., Cobbs S., Jordon J., Allison P. (2021). Process-structure-property relations for as-deposited solid-state additively manufactured high-strength aluminum alloy. Addit. Manuf..

[85] Phillips B.J., Williamson C.J., Kinser R.P., Jordon J.B., Doherty K.J., Allison P.G. (2021). Microstructural and mechanical characterization of additive friction stir-deposition of aluminum alloy 5083: Effect of lubrication on material anisotropy. Materials.

[86] Hartley W.D., Garcia D., Yoder J.K., Poczatek E., Forsmark J.H., Luckey S.G., Dillard D.A., Yu H.Z. (2021). Solid-state cladding on thin automotive sheet metals enabled by additive friction stir deposition. J. Mater. Process. Technol..

[87] Jordon J.B., Allison P.G., Phillips B.J., Avery D., Kinser R., Brewer L., Cox C., Doherty K. (2020). Direct recycling of machine chips through a novel solid-state additive manufacturing process. Mater. Des..

[88] Williams M.B., Robinson T.W., Williamson C.J., Kinser R.P., Ashmore N.A., Allison P.G., Jordon J.B. (2021). Elucidating the effect of additive friction stir deposition on the resulting microstructure and mechanical properties of magnesium alloy WE43. Metals.

[89] Joshi S.S., Patil S.M., Mazumder S., Sharma S., Riley D.A., Dowden S., Banerjee R., Dahotre N.B. (2022). Additive friction stir deposition of AZ31B magnesium alloy. J. Magnes. Alloys.

[90] Agrawal P., Haridas R.S., Agrawal P., Mishra R.S. (2022). Deformation based additive manufacturing of a metastable high entropy alloy via additive friction stir deposition. Addit. Manuf..

[91] Stubblefield G.G., Fraser K.A., Van Iderstine D., Mujahid S., Rhee H., Jordon J., Allison P. (2022). Elucidating the influence of temperature and strain rate on the mechanics of AFS-D through a combined experimental and computational approach. J. Mater. Process. Technol..

[92] Griffiths R.J., Gotawala N., Hahn G.D., Garcia D., Yu H.Z. (2022). Towards underwater additive manufacturing via additive friction stir deposition. Mater. Des..

[93] Agrawal P., Haridas R.S., Yadav S., Thapliyal S., Dhal A., Mishra R.S. (2023). Additive friction stir deposition of SS316: Effect of process parameters on microstructure evolution. Mater. Charact..

[94] Gotawala N., Yu H.Z. (2023). Material flow path and extreme thermomechanical processing history during additive friction stir deposition. J. Manuf. Process..

[95] Yoder J.K., Erb D.J., Henderson R., Yu H.Z. (2023). Closed-loop temperature controlled solid-state additive manufacturing of Ti-6Al-4V with forging standard out-of-plane tensile properties. J. Mater. Process. Technol..

[96] Beck S.C., Williamson C.J., Kinser R.P., Rutherford B., Williams M., Phillips B., Doherty K., Allison P., Jordon J. (2023). Examination of microstructure and mechanical properties of direct additive recycling for Al-Mg-Mn alloy machine chip waste. Mater. Des..

[97] Zhu Y., Wu X., Gotawala N., Higdon D.M., Yu H.Z. (2024). Thermal prediction of additive friction stir deposition through Bayesian learning-enabled explainable artificial intelligence. J. Manuf. Syst..

[98] Hahn G.D., Knight K.P., Gotawala N., Yu H.Z. (2024). Additive friction stir deposition of AA7050 achieving forging-like tensile properties. Mater. Sci. Eng. A.

[99] Costanza G., Montanari R., Quadrini F., Sili A. (2005). Influence of Ti coatings on the fatigue behaviour of Al-matrix MMCs. Part I: Fatigue tests and materials characterization. Compos. Part B Eng..

[100] Choudhury S., Acharya U., Sethi D., Roy J., Roy B.S. (2024). Synergic enhancement of ductility and toughness in friction stir additively fabricated AA6061-T6 build. J. Adhes. Sci. Technol..

[101] Soni V., Menchaca R.L., Davis D., Kumar N.N., Gonzalez M., Awasthi P., Haridas R.S., Loukus A., Weiss D., Mishra R.S. (2025). Process-specific design strategy enables exceptional as-deposited strength-ductility synergy in novel Al-Ce alloys via additive friction stir deposition (AFSD). J. Mater. Res. Technol..

[102] Qiao Q., Wang L., Zhu Z., Lin Y., Fu K., Qian H., Li Z., Guo D., Zhang D., Kwok C. (2025). New method for fabricating 6061-7075-composite with enhanced microstructure, mechanical properties, and electrochemical resistance using additive friction stir deposition and heat treatment. Mater. Sci. Eng. A.

[103] Zavari S., Bagheri E., Ding H., Adibi N., Eller M., Cox C., Guo S. (2025). The influence of additive friction stir deposition process on mechanical properties, corrosion resistance, and electrical conductivity of Al5086-H32 alloy. Prog. Addit. Manuf..

[104] Bagheri E., Adibi N., Wilson P., Talachian M., Ding H., Khonsari M., Guo S. (2026). Corrosion resistance and fatigue life of Al 7075 parts prepared by high-rate additive friction stir deposition. J. Mater. Eng. Perform..

[105] Tuo Z., Chen J., Liu Z., Ren J., Ye J., Zheng Z., Wu H. (2026). Effect of initial feedstock conditions on microstructure and mechanical properties of additive friction stir deposited Al-Cu-Mg-Ag alloy. J. Mater. Res. Technol..

